# Biomarker and isotopic characteristics of Miocene condensates and natural gases, West Delta deep marine concession, Eastern Mediterranean, Egypt

**DOI:** 10.1038/s41598-023-50418-4

**Published:** 2024-01-02

**Authors:** Naira M. Lotfy, Sherif Farouk, Mohammed Hail Hakimi, Fayez Ahmad, Tamer El Shennawy, Mohamed M. El Nady, Ahmad Salama, Amr M. Shehata

**Affiliations:** 1 Exploration Department, Egyptian Petroleum Research Institute (EPRI), Ahmed El Zommor St., Nasr City, Cairo, 11727 Egypt; 2 Geology Department, Faculty of Applied Science, Taiz University, 6803 Taiz, Yemen; 3 Prince El-Hassan Bin Talal Faculty of Natural Resources and Environment, Department of Earth and Environmental Sciences, The Hashemite University, Zarqa, 13115 Jordan; 4 Rashid Petroleum Company, New Maadi Cairo, 11742 Egypt; 5 Egyptian General Petroleum Corporation, Cairo, Egypt

**Keywords:** Geochemistry, Geology

## Abstract

The Western Delta Deep Marine Concession (WDDM) in the Eastern Mediterranean Sea is one of northern Africa's most recent petroleum-potential regions for gas and condensate exploration. The present study aims to determine the characteristics of the 15 natural gases and 5 associated condensate samples, using molecular compositions and isotopes from the Miocene reservoir rocks in the various wells located in the WDDM. The results of this study are also used to determine the gas-condensate correlation for their probable source rocks as well as the methane-generating mechanisms (i.e., thermogenic or microbiological). Results highlighted in this research reveal that most of the natural gases in WDDM are mainly thermogenic methane gases, with small contributions of biogenic methane gases that were generated from mainly mixed sources, with a high sapropelic organic matter input for biogenic gases. The thermogenic methane gases were formed from secondary oil and oil/gas cracking at the high maturity stage of the gas window. The biogenic gases are also contributed to the Miocene reservoirs, which are formed from the primary cracking of kerogen at low maturity stage by the action of CO_2_ bacterial reduction. In addition, the saturated and aromatic biomarker results show that the condensate samples were generated from clay-rich source rocks. This source unit of the Miocene condensates were deposited in a fluvial deltaic environmental setting, containing mixed kerogen type II/III and accumulated during the Jurassic–Cretaceous, as evidenced by the age dating indicators. The properties of the natural gases and associated condensates in the Miocene reservoir rocks suggest that most of the thermogenic methane gases, together with the condensate, are derived primarily from mature Jurassic–Cretaceous source rocks and formed by secondary oil and oil/gas cracking at the gas generation window, as demonstrated by the 1-D basin modelling results highlighted in the prior works. Therefore, most of the natural gases in WDDM are non-indigenous and migrated from more mature Jurassic–Cretaceous source rocks in the nearby Northern Sinai provinces or the deeper sequences in the offshore Nile Delta provinces.

## Introduction

**Figure 1:**
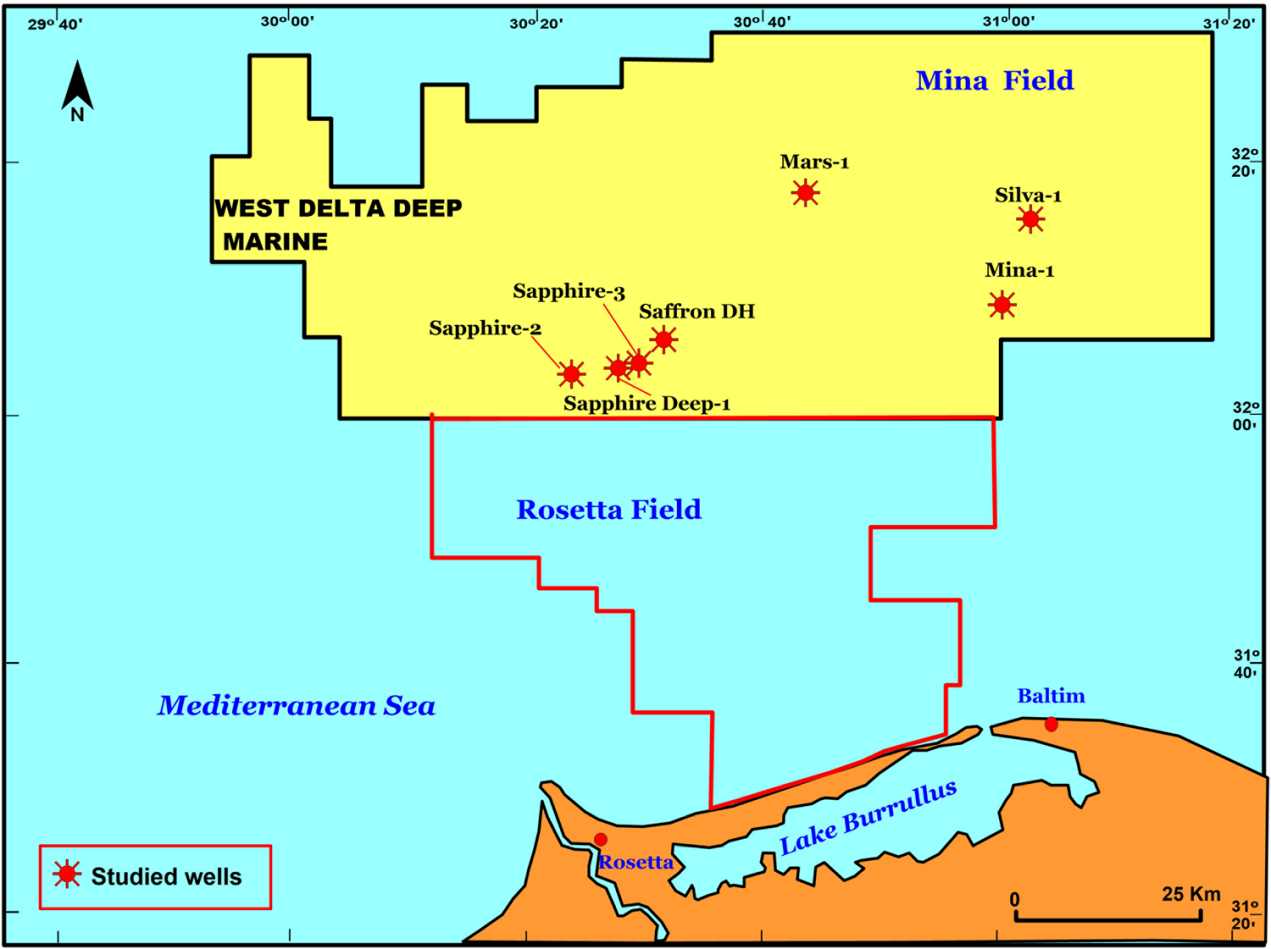
Location map of the studied wells in the Nile Delta off region, WDDM (Golden.Software.Surfer.v13.3.493. × 86. × 64; Serial WS-172883-98ac).

The western offshore Nile Delta is one of the most promising and productive regions in the Eastern Mediterranean and adds the most gas to Egypt's reserves after recently discovering the Zohr gas field (e.g.^1,2^). Recently, the exploration within the WDDM block is focused on a number of deep plays and prospects. The deep wells within WDDM have targeted Serravalian plays (Sapphire Deep-1) and Messinian plays (Mars-1and Mina-1) (Fig. 1).

The petroleum system in the western offshore Nile Delta is unclear despite recent big oil and gas finds, since few wells have reached the pre-Miocene succession^2^. Many studies have been focused on studying the geochemical characteristics of the Pliocene-Miocene formations due to the limited samples of Pre-Miocene formations. These studies prevailed that the Pliocene succession is primarily immature in the Nile Delta basin, and the Miocene succession only reached to the early stage of oil generation^3,4^. In addition, The Mesozoic formations that correspond to the Khatatba Formation (mid-Jurassic), Upper Cretaceous Abu Roash and Khoman formations are considered to be the main source rocks for both oil and gas generation potential in the northern Western Desert^5,6^. These formations are anticipated to be buried deep beneath the surface in the offshore of northern Egypt, and are expected to be the main source rocks for both condensate and gas generation potential in the Nile Delta basin. However, it is still unclear how much these deep intervals contribute to the production of hydrocarbons in the Nile Delta.

There have been few geochemical investigations of the Nile Delta sedimentary succession's source rocks' potential and maturity, sources of natural gas accumulations and condensate accumulations, and related topics (e.g.^3,4,7,8^). Recent studies documented that there is no correlation between condensates and Upper Cretaceous rock extracts^2,9^. In this regard, the current study attempts to give further important geochemical details regarding the intricate processes by which the hydrocarbons (gas and condensate) are supplied, charged, mixed, and transformed in WDDM.

## Geological settings

Since the Late Miocene, the Messinian Salinity Crisis has been primarily responsible for the formation of the deep-sea fan of the Nile Delta^10^. As a result of the tectonically caused constriction of the western link to the Atlantic Ocean, climatic circumstances, sea level changes, and evaporites were formed all over the Mediterranean concession^11^. Contraction deformation resulted in raised arches and nearby strike-slip grabens in the Nile Delta.

Fluvial incisions through the continental shelf the size of the Grand Canyon were caused by synchronised sea-level drops^12^. As a result, large amounts of terrigenous sediments were carried offshore by the proto-Nile river into the Eastern Mediterranean concession^13^. The continued deposition of mixed fluvial-deltaic sediments has resulted in the partial erosion of the evaporitic deposits since the Early Pliocene transgression^14^. The thickness of the Nile delta sediments is estimated to be 9–10 km, ranging in age from Jurassic to Quaternary^15^.

**Figure 2:**
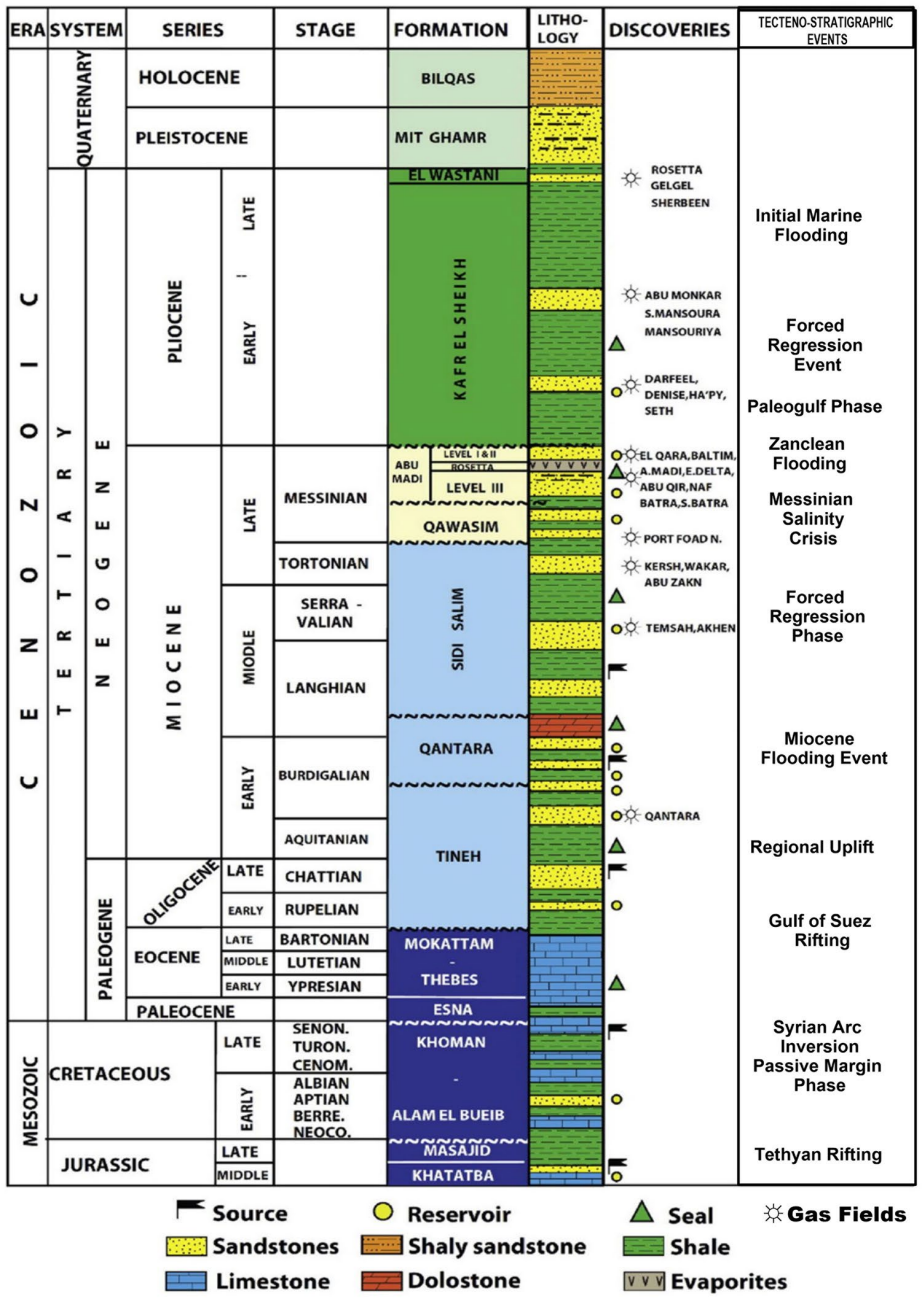
Stratigraphic column of Nile Delta^20^.

The Jurassic period marks the end of the penetrated sedimentary succession in the Nile Delta (Fig. 2). The Neogene-Quaternary clastics represent the rock units that have petroleum potential^16,17^. The Upper Jurassic formations are the oldest rock units in the area^15^. During the Upper Cretaceous, the deposition of marine-alluvial sediments predominated^18,19^. Because of Syrian Arc Folding (SAF), the Late Cretaceous and Eocene units are thin^12^. Siliciclastic fluvial facies of high thickness have represented Oligocene rock units^12,18^. In Miocene-Pliocene sequences, two notable unconformities are recorded separated by the Messinian Salinity Crisis desiccation event, which resulted in the formation of Abu Madi incisions^12,18,20^. Marine facies which have represented in Kafr El-Sheikh Formation, covered the Nile Delta basin during the Lower Pliocene^18,21,22^ (Fig. 2).

## Samples and analytical methods

In the current study, fifteen natural gas and five condensate samples from the Miocene reservoir rocks were collected from wells ( Mina-1, Sapphire deep-1, Sapphire-2, Sapphire-3, Mars-1 and Silva-1) located in the Serravalian and Messinian plays (Fig. 1). Geochemical analyses were performed on these samples of natural gases and condensate. .

### Chemical analyses of gases

Gas molecular and isotopic compositions were performed at stratochem laboratories (Cairo, Egypt). In this regard, components of individual hydrocarbon gases (C_1_–C_5_), N_2_, O_2_ and CO_2_ gases (permanent gases) were separated using three capillary and packed columns, respectively. Both capillary and packed columns are connected to one oven, whose temperature is raised from 50 to 180 °C at a rate 10 °C/min.

In addition, the carbon (δ^13^C) and hydrogen (δD) isotopic compositions of hydrocarbon gases and non-hydrocarbon gases (δ^13^C-CO2) were detected with a TFDX-MS (Thermo Finnigan Deltaplus XL mass spectrometer). A gas chromatograph was used to separate the gas components, which were then injected into the MS (Mass Spectrometer). Isotope values are reported in per-mile units (‰) attributed to the PDB (common Pee Dee Belemnite) and SMOW (Vienna Standard Mean Ocean Water) standards. (The analytical precision of δ^13^C and δ D measurements is approximately ± 0.2‰ and ± 2‰, respectively)^23,24^.

### Geochemical analyses of condensates

Condensate samples were examined by a number of geochemical studies, including fractionation, gas chromatography (GC), gas chromatography-mass spectrometry (GC–MS) and carbon (δ^13^C) isotope. The procedures for these analyses are highlighted in the following:

The whole condensate samples were injected into a gas chromatograph (Varian 3400) with a 100-m capillary column. The oven temperature remains constant at 35 °C until detecting the concentration of C_2_–C_8_ compounds^25^.

The condensate samples were subsequently fractionated into different fractions (saturates, aromatics) by successive hexane and benzene elutions on the silica filled gravity column. The saturate and aromatic fractions are subjected to further analyses. A Hewlett-Packard 5890 gas chromatograph and a capillary column (Quadrex 50-m) are used to analyse the saturate fraction. The samples were heated from 40 to 340 °C, at a rate of 10 °C/min and went through the GC line operating at 340 °C for 20 min.

Later, the GC–MS was utilized to decipher the saturated and aromatic hydrocarbon fractions within the analyzed condensate sample using a HP 5890 GC linked to MSD (Mass Selective Detector). The GC is programmed from 40 to 340 °C at 10 °C/min with a hold at 40 °C and a 20-min hold at 340 °C. As a result, the biomarker compounds within the saturated and aromatic HC fractions i.e., hopanoid, terpane, sterane, naphthalene, and phenanthrene groups were produced and analyzed based on the peak integration using a Hewlett-Packard chemstation data acquisition system and IBM computer.

## Results and interpretation

### Molecular and isotope compositions of natural gases

The molecular compositions of studied natural gases show that most of the samples are dominance by methane content in the range of 86.53–98.13%, except one gas sample from Mina-1 well has low methane content with value equal to 28.55% (Table 1). These natural gases also have heavy gas components (C_2_–C_5_) in low concentrations of less than 5% (Table 1). In addition, the dominant non hydrocarbon gases i.e. N_2_, O_2_ and CO_2_ are also presented with low amounts for most of the analyzed natural gases, except one sample from Mina-1 well, with low methane content contains a high concentration of nitrogen gas with value reaches to 69.6% (Table 1).

**Table 1:** Molecular composition and stable carbon and hydrogen isotopic compositions of gas samples from different wells in west delta deep marine concession.

Well name	Depth (m)	Age	Hydrocarbon gas composition (mol%)							
			CH_4_	C_2_H_6_	C_3_H_8_	iC_4_H_10_	n-C_4_H_10_	iC_5_H_12_	n-C_5_H_12_	n-C_6_H_14_
Mina-1	3595	Miocene	91.1	4.14	1.32	0.29	0.34	0.14	0.11	0.31
	3610		86.94	4.14	1.46	0.35	0.44	0.22	0.17	0.58
	3723.5		28.55	0.52	0.1	0.01	0.02	0.01	0.01	0.02
Mars	4278		87.85	0.10	0.05	0.03	0.03	0.04	0.02	0.12
Sapphire Deep-1	2598.5		94.30	4.40	1.12	0.07	0.05	0.02	0.03	0.01
	2744.5		95.22	3.68	0.97	0.07	0.04	0.01	0.00	0.00
	2777.2		95.72	3.32	0.50	0.13	0.11	0.07	0.06	0.09
	4105.5		98.13	1.06	0.68	0.03	0.03	0.02	0.02	0.03
	4216.5		98.01	1.33	0.49	0.02	0.02	0.01	0.09	0.02
Saffron DH	2009.00		93.51	2.22	0.35	0.19	0.13	0.09	0.06	0.15
Sapphire-3	2598.50		92.32	3.08	1.14	0.31	0.30	0.14	0.10	0.30
	2839.60		91.07	3.83	1.23	0.31	0.31	0.16	0.11	0.43
Silva-1	2940.50		87.73	6.38	2.66	0.70	0.69	0.31	0.23	0.58
	3129.50		86.97	6.56	2.85	0.76	0.76	0.34	0.25	0.62
	3181.20		86.53	6.65	2.90	0.77	0.79	0.36	0.27	0.67

Well name	Non-hydrocarbon gas composition (mol %)		Gas dryness (%)	Gas wetness (%)	Stable carbon and hydrogen isotopic compositions					
					δ^13^C (‰, VPDB)				δ^2^H(‰,VSMOW)	
	CO_2_	N_2+_ O_2_			δ^13^C_1_	δ^13^C_2_	δ^13^C_3_	δ^13^CCO_2_	δ^2^H-C_1_ (δ D- C_1_)	
Mina-1	0.63	1.63	93	7	− 41.41	− 27.20	− 26.20	− 9.9	− 155.30	
	0.52	5.17	93	7	− 41.37	− 27.00	− 26.30	− 8.9	− 163.90	
	1.17	69.6	98	2	− 46.41	− 27.67	− 26.39	− 13.05	− 165.00	
Mars	2.80	8.97	100	0	− 59.56	− 27.60	− 27.20	N.D	− 177.10	
Sapphire Deep-1	N.D	N.D	94	6	− 47.23	− 28.40	− 26.50	N.D	− 162.91	
	N.D	N.D	95	5	− 47.23	− 29.40	− 26.50	N.D	− 159.10	
	N.D	N.D	96	4	− 45.62	− 30.10	− 25.20	N.D	− 159.10	
	N.D	N.D	98	2	− 53.67	− 28.30	− 26.00	N.D	− 167.68	
	N.D	N.D	98	2	− 46.03	− 27.60	− 25.20	N.D	− 157.19	
Saffron DH	0.14	3.16	97	3	− 51.44	− 29.84	− 27.8	N.D	− 176.8	
Sapphire-3	0.16	2.15	95	5	− 51.67	− 30.1	− 27.4	N.D	− 176.3	
	0.26	2.29	94	6	− 44.32	− 29.61	− 26.82	N.D	− 161.6	
Silva-1	0.27	0.44	89	12	− 48.63	− 29.7	− 26.3	− 14.3	− 168.6	
	0.30	0.59	88	12	− 48.45	− 29.61	− 26.45	− 14.03	− 166.2	
	0.35	0.75	88	12	− 48.7	− 29.68	− 26.26	− 14.57	− 168.8	

Dryness index (%) = 100*C_1_/(∑C_1_–C_5_).

Wetness index (%) = 100*∑ (C_2_–C_5_)/(∑C1–C_5_).

The dryness and wetness indices of the studied gases are also calculated based on the hydrocarbon gases (C_1_–C_5_), listed in Table 1. The dryness and wetness values of the samples studied range widely. Gases from Mars-1 well display very high dryness value that reached to 100%, while the gas samples from other wells display relatively low dryness values ranging between 88 and 98% (Table 1). However, most of the studied gases (70%) have relatively high wetness index of more than 5%, classifying as wet gases according to^26^**.**

**Figure 3:**
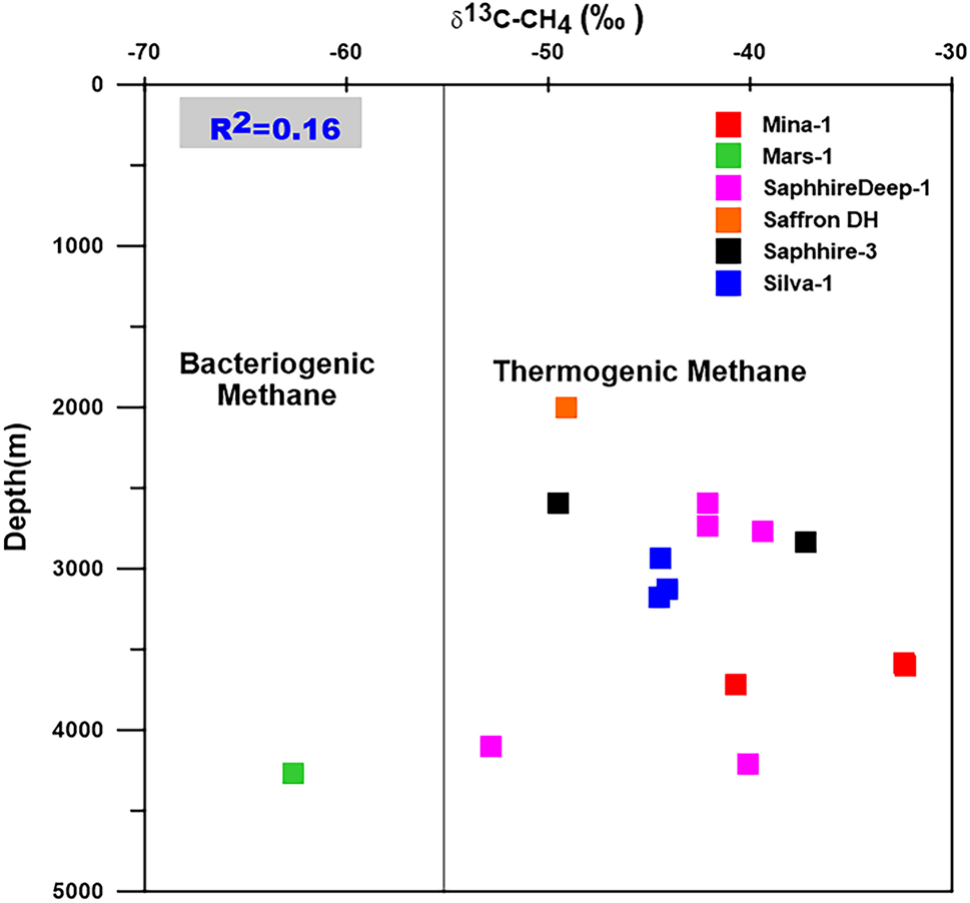
Relation between depth and δ^13^C (‰) of the WDDM gases.

The isotope compositions of the studied gases show that the methane carbon (δ^13^C-CH_4_) and hydrogen isotope compositions (δ^2^H-CH_4_) range from − 51.67 to − 41.37‰ and − 176.8 to − 155.3‰, respectively. The relation between isotope composition and depths of the studied samples is applied and shows that the lighter values (more negative) are presented in two deeper Miocene samples at depths of 4105.5 m and 4278 m. They have methane carbon (δ^13^C-CH_4_) and hydrogen isotope values equal to − 53.67‰ and − 167.68‰ respectively, at depth 4105.5 m, and − 59.56‰ and − 177.10‰ at depth 4278m (Table 1). Most of the analyzed gas samples have methane carbon (δ^13^C-CH_4_) isotopes of more than − 55‰ indicate thermogenic methane (Fig. 3). However, the general increase in the δ^13^C-CH_4_ values with depth is not observed in the studied gas samples, suggesting that these gases have migrated to the current reservoir intervals and are not native (Table 1)^27,28^.

The stable carbon isotopic distribution of methane (CH_4_) and its homologues (C_2–5_) in the studied wells are characterized by a normal pattern (δ^13^C-CH_4_ < δ^13^C-C_2_H_5_ < δ^13^C-C_3_H_6_) (Fig. 3). The studied samples have heavier isotopic compositions of ethane and propane, ranging from – 30.10‰ to – 27.00‰ and – 27.80‰ to – 25.20‰ (Table 1). The δ^13^C-CH_4_ values show that most of the natural gases from the studied wells in WDDM are mainly thermogenic methane gases, except for one sample that is bacteriogenic (Fig. 3).

**Figure 4:**
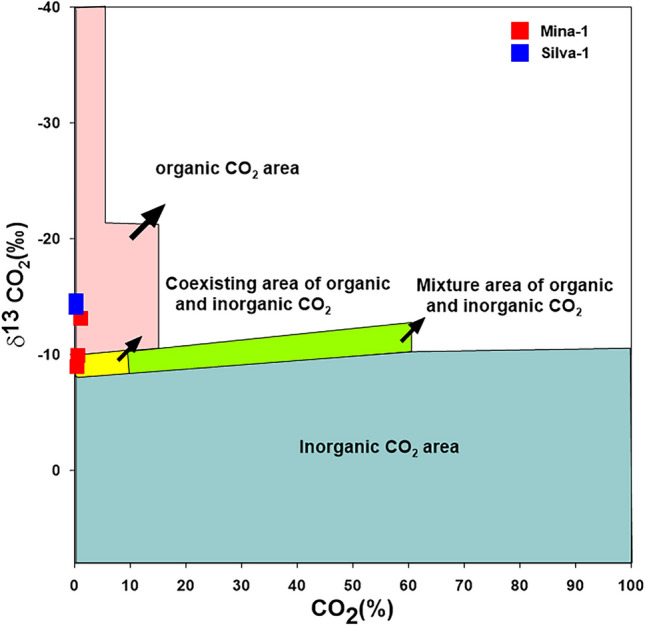
Origin of CO_2_ based on the relation between CO_2_ and δ^13^CO_2_.

In addition, the isotopic values of non-hydrocarbon gases (CO_2_) are measured for some gas samples. Accordingly, the δ^13^C-CO_2_ values range between – 14.57 and – 8.9‰ with an average content of – 12.46‰ (Table 1). The CO_2_ gas may result from the thermal cracking of organic matters, decomposition of marine carbonates, atmosphere, and mantle degassing^29^. In our case, the CO_2_ gas mainly of thermogenic origin and formed from the thermal decomposition of organic matter at a high maturity stage (Fig. 4).

### Light hydrocarbon composition (C_5_–C_7_) of condensate

**Figure 5:**
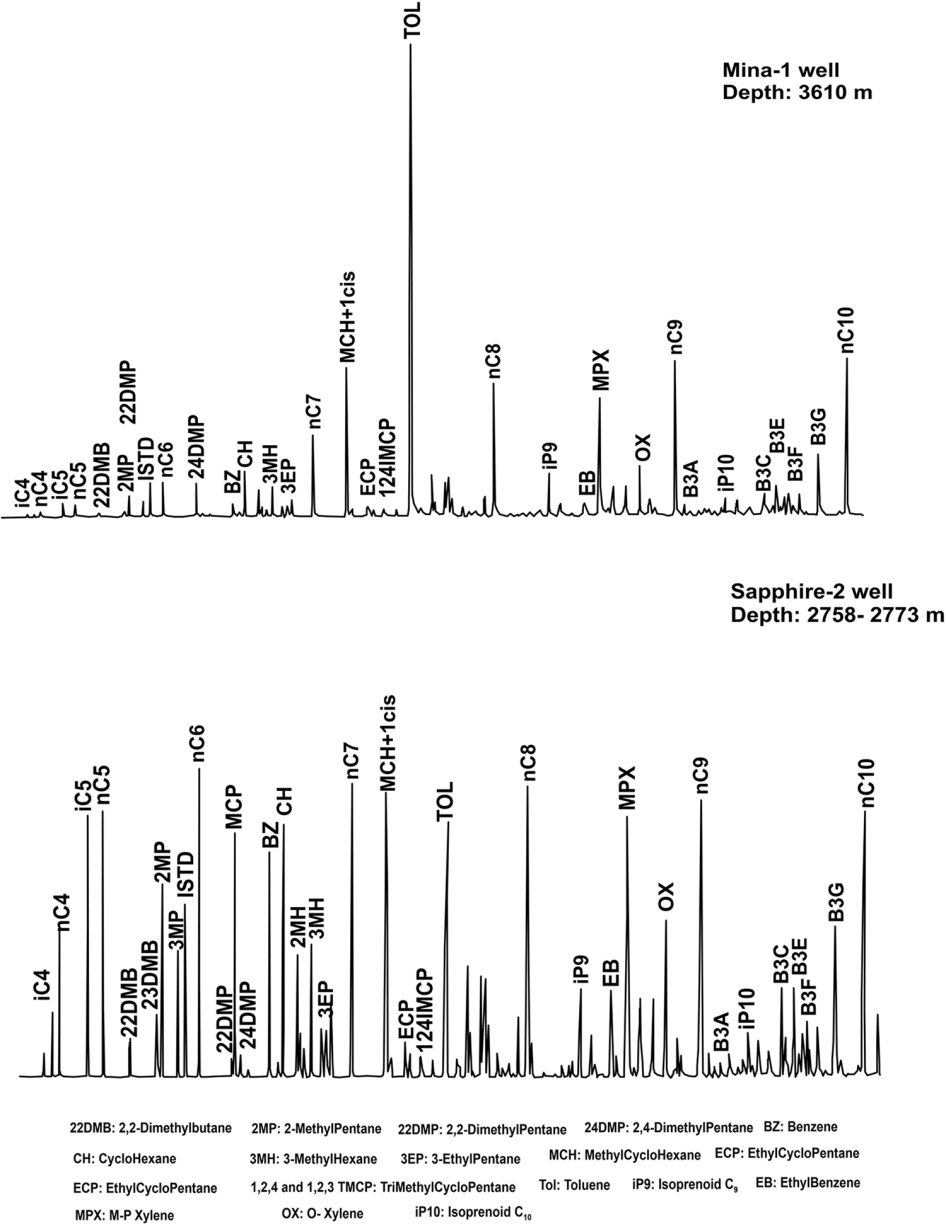
Gas chromatogram of C_5_–C_7_ light hydrocarbons of representative condensate samples.

The light hydrocarbon compounds (C_5_–C_7_) of the condensate samples associated with wet gases are presented in Fig. 5 and Table 2. The results show a high abundance of toluene and methylcyclohexane (MCH) compared to normal alkanes in some condensates (Mina-1 and Sapphire-3 wells) (Fig. 5; Table 2). These condensates display high aromaticity values (Toluene/n-C_7_) ranging between 2.05 and 6.50, indicating that some condensates have suffered from the evaporative fractionation process during migration^30^.

**Table 2:** Light hydrocarbon parameters, isotopic compositions, bulk and gas chromatography results for one condensate sample from Mina-1 well in the study area.

Well name	Depth (m)	Thompson light HC ratios		Bulk composition			Isotopic composition			Gas chromatography parameters		
		Toluene/nC-_7_	n-C_7_/MCH	Saturate %	Aromatic %	NSO compounds %	δ^13^C saturates	δ^13^C aromatics	CV	Pr∕Ph	Pr∕n-C17	Ph∕n-C18
Mina-1	3160	6.50	0.56	81.10	15.32	3.58	− 26.30	− 24.60	0.28	3.20	0.39	0.15
Sapphire-2	2758–2773	0.77	0.56	75.20	21.20	3.60	− 27.93	− 25.65	2.07	4.76	1.04	0.31
Sapphire-3	2839.6	2.05	0.60	75.27	22.26	2.47	− 28.10	− 25.80	2.17	5.83	0.71	0.18
Silva-1	2817	1.03	0.54	84.64	13.17	2.20	− 27.00	− 23.85	3.71	3.69	0.26	0.10
	3181.2	0.77	0.58	85.82	9.54	4.64	− 27.30	− 24.05	4.03	5.30	0.30	0.08

*MCH* methyl cyclohexane, *CV* canonical value.

### Bulk geochemical and molecular composition of condensate

Saturates, aromatics, and polar components of the condensate samples were separated, and their relative amounts were determined and are shown in Table 2. The saturated HC fraction dominates over the aromatic fraction (Table 2). Such compositions are typical of highly mature condensates derived from source rocks with high maturity levels. In addition to the HC fractions, the condensate sample contains low amounts of polar components (Table 2).

**Figure 6:**
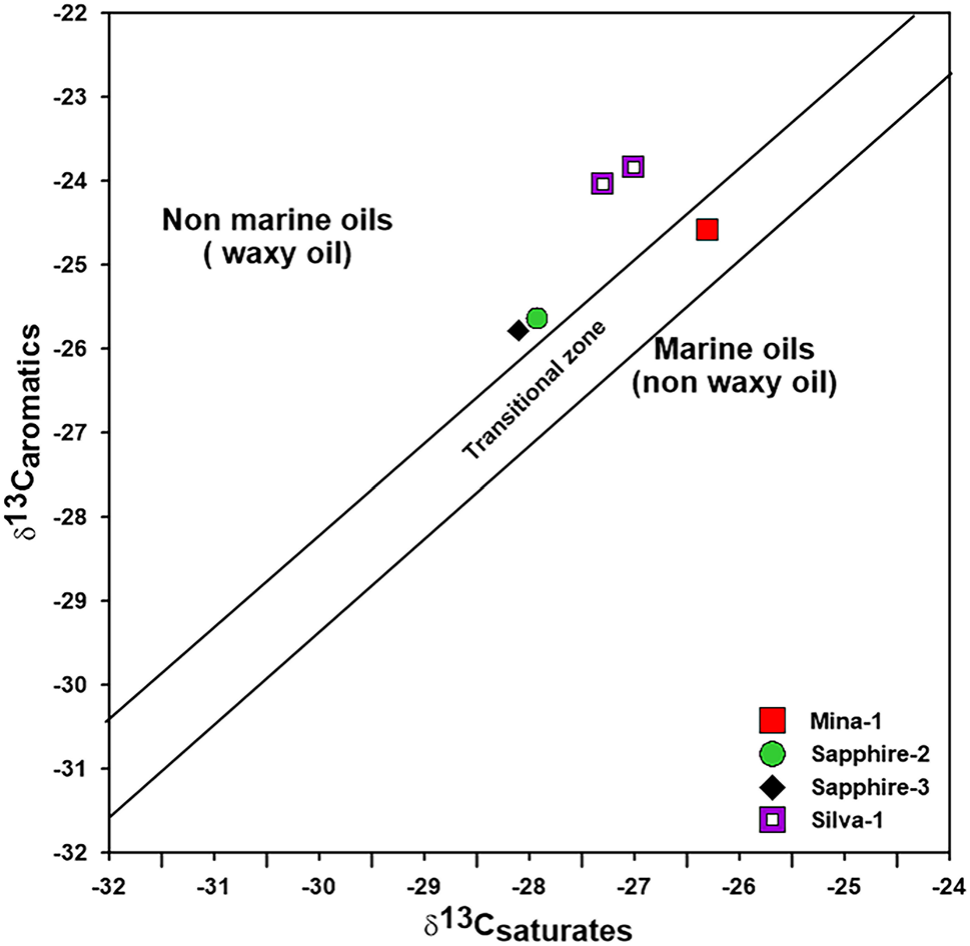
Sofer isotope plot for the studied condensate samples.

In this study, bulk stable δ^13^C composition was measured for the aliphatic and aromatic hydrocarbon fractions from the examined Miocene condensates, and the results were tabulated in Table 2. Values of δ^13^C for the saturate and aromatic fractions range between – 28.10‰ and – 26.30‰, and – 25.80‰ and – 23.85‰ respectively, with canonical variable (CV) values between 0.28 and 4.03‰ (Table 2). According to several scientists, the isotopic hydrocarbon fractions are critical in distinguishing terrigenous organic matter from marine organic matter. Low δ^13^C values indicate terrigenous origin, whereas high and moderate δ^13^C values indicate aquatic algae and microbes^31,32^. As a result, the examined samples are primarily derived from different depositional environments, as evidenced by the saturated (δ^13^C_Sat_) and aromatic (δ^13^C_Aro_) isotope compositions displayed on the diagram of^31^ (Fig. 6).

**Figure 7:**
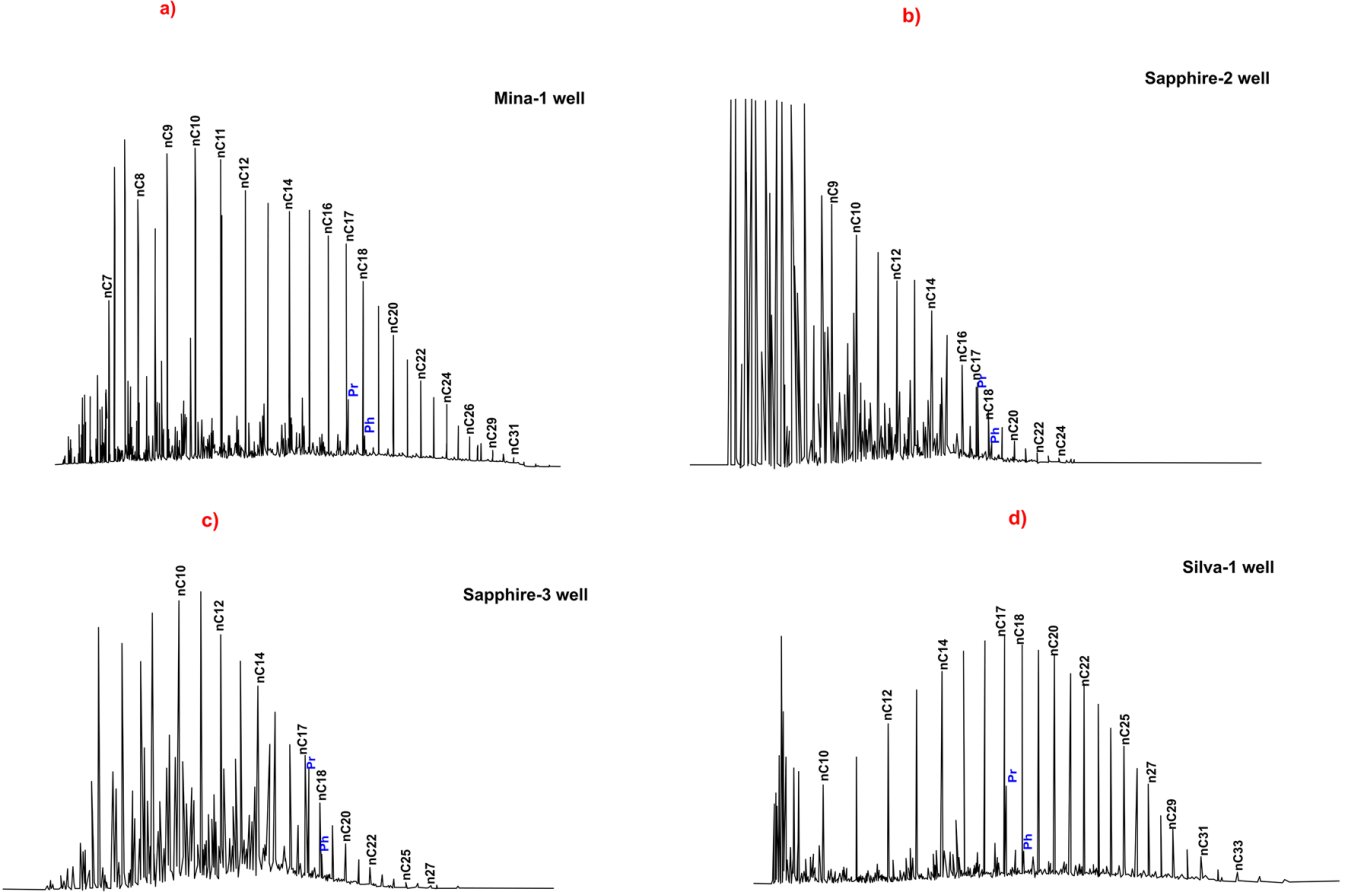
Gas chromatograms of the studied condensate samples.

A GC profile was used to reveal the hydrocarbon distributions of *n*-alkanes, pristane (Pr), and phytane (Ph) in the investigated Miocene condensates, which display a unimodal distribution of *n*-alkanes with low molecular weight (< n-C_20_) with a slight bias towards odd/even n-alkanes (Fig. 7). Low abundance of normal alkanes (> n-C_21_) is observed in the studied samples (long chain), and this may be related to the condensates with high maturity stages (Fig. 7; Table 2)^2^.

In the chromatograms, Pr predominated over Ph for the condensate samples (Fig. 7), leading to comparatively high Pr/Ph ratios (3.2–5.83). The studied condensate samples also have low Pr/*n*-C_17_ and Ph/*n*-C_18_ ratios, with values 0.39–1.04 and 0.08–0.31, respectively (Table 2).

### Biomarker fingerprints of condensate

**Figure 8:**
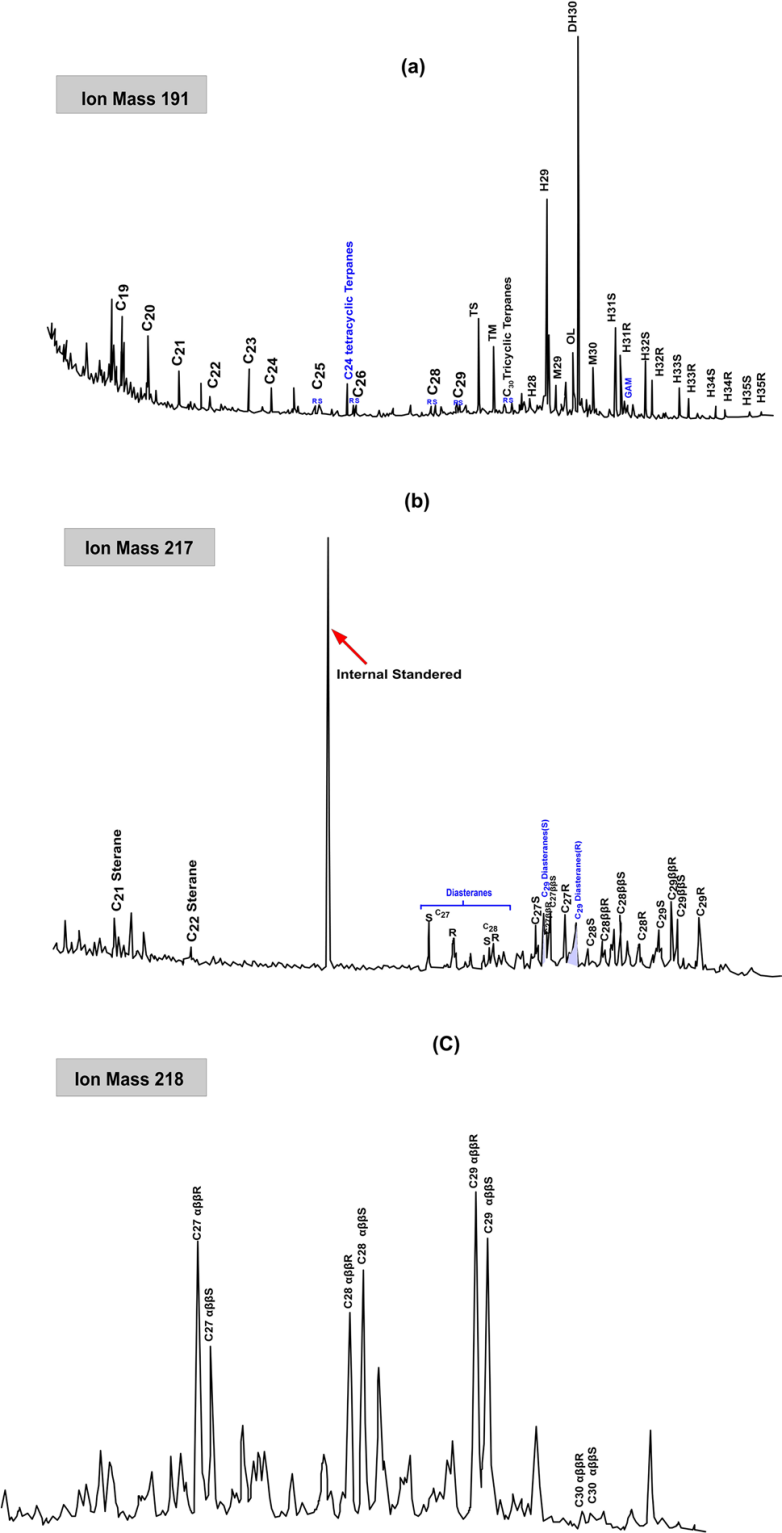
Mass chromatogram of m/z 191 (terpanes), 217 and 218 (steranes) of the representative condensate sample.

Terpanes and steranes biomarker distributions in the saturated fraction of the representative Miocene condensate were examined by MS of *m/z* 191, 217, and 218 ions (Fig. 8).

The mass fragmentogram (m/z 191) shows that pentacyclic terpanes (hopanes) are more abundant than tricyclic terpanes. (Fig. 8a). C_30_ hopanes are more prevalent than other hopanes. The greater frequency of C_30_ hopane leads to a low C_29_ norhopane/C_30_ hopane value of less than 1 (Table 3), indicating that this condensate is generated from clay-rich source rock^33^. This conclusion is supported by the distribution of C_31_ homohopanes, which shows that the C_31_ homohopanes predominate in comparison to the other homohopanes (C_31_ > C_32_ > C_33_ > C_34_ > C_35_; Fig. 8a) (Table 3). A substantial amount of C_30_-oleanane is noted in the studied sample (Fig. 8a), with Oleanane/C_30_ hopane values ranging between 0.09 and 0.24 (Table 3). In addition, minor amounts of gammacerane and diahopanes have been detected, yielding low gammacerane/C_30_ hopane and diahopane/C_30_ hopane ratios, respectively (Table 3).

**Table 3:** Saturated biomarker ratios of condensate samples from different wells in the WDDM concession.

Well name	Depth (m)	Steranes												Terpanes							
		%C27 αββS Sterane	%C28 αββS Sterane	%C29 αββS Sterane	%C27 ααα Sterane	%C28 ααα Sterane	%C29 ααα Sterane	C29 S/(S + R)	C29αββ/(αββ + ααα)	Diasterane/ααα Sterane	C28/C29 αββS	C30 αββS Sterane Index (218)	Ol/Hopane	Norhopane/Hopane	Diahopane/Hopane	C22/C21 Tricyclic	C24/C23 Tricyclic	C26/C25 Tricyclic	C35/C34 homohopanes	Steranes/Hopanes	C32 22S/(22S + 22R) homohopanes
Mina-1	3160	27.08	32.08	40.84	31.38	24.77	43.85	0.44	0.52	0.76	0.80	1.99	0.23	0.56	0.07	0.39	0.59	1.22	0.47	0.36	0.59
Sapphire-2	2758–2773	29.12	31.01	39.87	40.84	22.44	36.71	0.37	0.43	0.63	0.80	–	0.23	0.57	0.06	0.28	0.53	0.94	0.84	0.32	ND
Sapphire-3	2839.6	–	–	–	32.23	23.46	44.31	0.33	0.51	1.32	0.00	–	0.24	0.63	0.06	0.26	0.63	1.00	0.68	0.37	ND
Silva-1	2817	19.00	30.41	50.59	25.74	27.31	46.94	0.37	0.51	1.38	0.60	3.11	0.10	0.53	0.18	0.44	0.46	1.03	0.38	0.36	ND
	3181.2	28.87	33.57	37.56	24.28	21.65	54.07	0.32	0.49	2.62	0.90	2.35	0.09	0.62	0.14	0.38	0.49	0.93	0.73	0.44	ND

The tricyclic terpane ratios in the analysed sample were also calculated and listed in Table 3. The analysed samples show high ratios of C_26_/C_25_ tricyclic terpanes and low ratios of C_22_/C_21_ and C_24_/C_23_ tricyclic terpanes (Table 3).

The biomarker distribution of steranes and their interpretative ratios were also identified using *m/z* 217 and 218 mass fragmentograms (Fig. 8b,c; Table 3). C_29_ sterane is the most predominant of the C_27_–C_29_ sterane series in the m/z 217 and m/z 218 ions for the studied samples (Fig. 8b,c), there is a significant amounts of the C_27_ and C_28_ steranes, with the percentages of C_27_, C_28_ and C_29_ regular sterane in the range of 24.28–40.84%, 22.44–27.31% and 36.71–54.07% respectively (Table 3).

**Figure 9:**
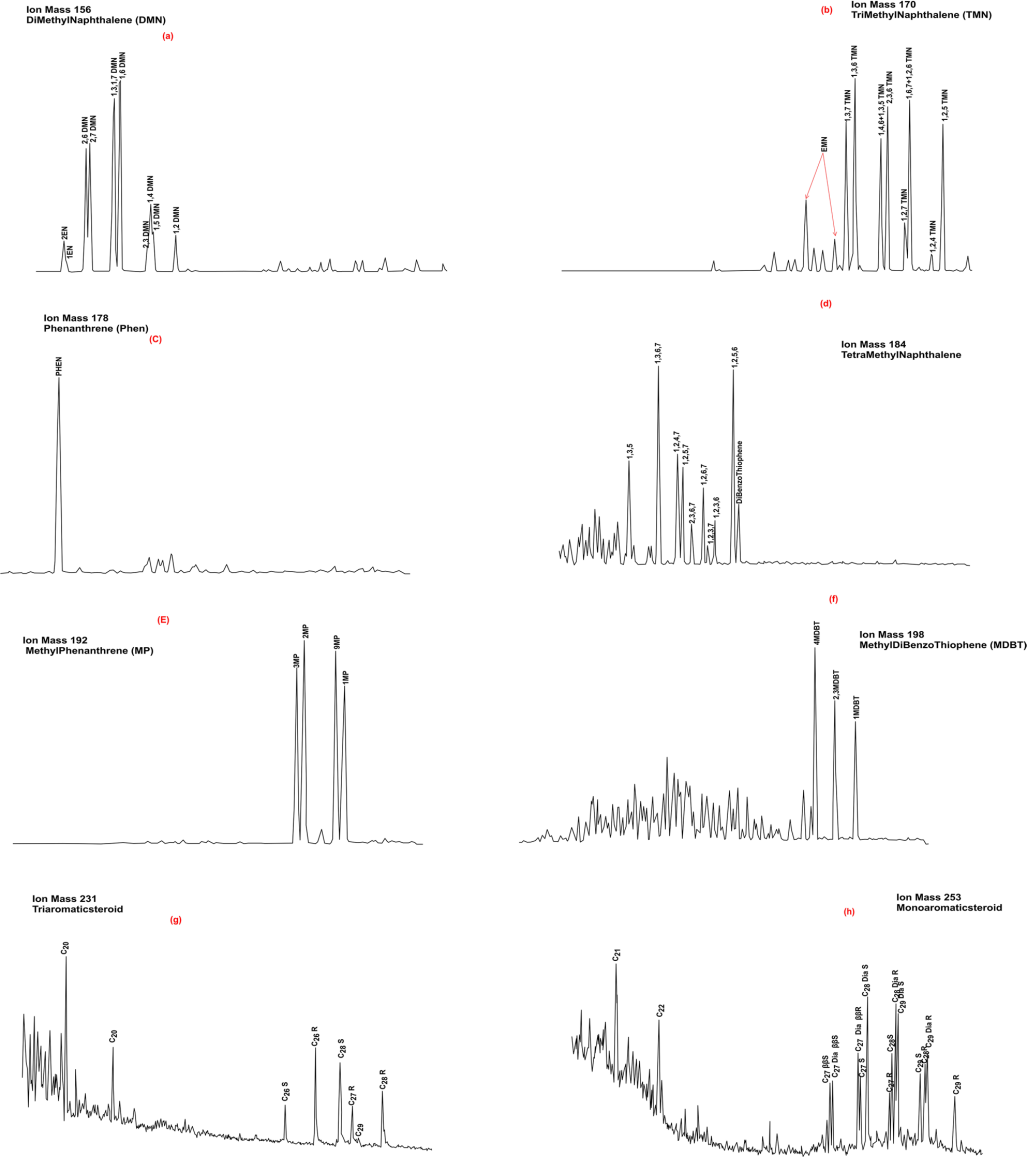
Mass chromatograms of m/z 156, 170, 178, 184, 192, 198, 231 and 253 illustrating the distribution of different aromatic compounds in condensate sample.

In the current study, the aromatic compounds from the analyzed condensate sample, such as Methyl-Naphthalene (Di-tri and tetra) (MN), Phenanthrene (P) Methyldibenzothiophene, Monoaromatic (MAS), triaromatic (TAS) and triaromatic methyl steroids (TAMS) were identified in the aromatic fractions using *m/z* 156, 170, 184, 178, 192, 206, 198, 253, 231 and 245 mass fragmentograms, respectively (Fig. 9). The representative ratios of these used aromatic compounds were calculated and listed in Table 4.

**Table 4:** Aromatic biomarker ratios of condensate samples from different wells in the WDDM concession.

Aromatic compounds	Interpretative ratios	Mina-1 (3610 m)	Sapphire-2 (2758–2773 m)	Sapphire-3 (2839.6 m)	Silva-1 (2817 m)	Silva-1 (3181.2 m)
Naphthalenes	DNR-1	7.96	–	–	–	–
	DNR-2	2.57	–	–	–	–
	TNR-1	1.06	–	–	–	–
	TDE (1,2,7- TMN/1,2,6-TMN)	0.25	–	–	–	–
	1,2,7-TMN/1,3,7-TMN	0.32	–	–	–	–
	1,2,5-TMN/1,3,6-TMN	0.66	–	–	–	–
	MNR	1.6	–	–	–	–
Phenanthrenes	1-MP/9-MP	0.83	–	–	–	–
	MPI-1	0.70	0.92	0.98	0.78	0.83
	MPI-2	0.74	–	–	–	–
	MPI-3	1.07	–	–	–	–
	MDR	1.56	–	–	–	–
	Rc(a) (Ro < 1.3)	0.79	–	–	–	–
	Rc(k)	1	–	–	–	–
	3MBP/3 + 4MBP	0.7	–	–	–	–
Triaromatic Steroids	C20 + C21)/Σ TAS	0.36	0.26	0.30	0.59	0.49
	TAS C21/C21 + C28	0.51	–	–	–	–
	% C26 TAS	22.1	–	–	–	–
	% C27 TAS	35.8	–	–	–	–
	% C28 TAS	42.1	–	–	–	–
	TAS C28/C26(20S)	3.2	–	–	–	–
	TAS C28/C27(20R)	1.17	–	–	–	–
Monoaromatic Steroids	% C27 MAS	26.70	27.14	25.00	28.62	26.78
	% C28 MAS	40.4	32.14	41.52	39.72	39.56
	% C29 MAS	32.9	40.72	33.48	31.67	33.65
Sulfur compounds	DBT/Phenanthrene	0.1	1.64	0.11	0.10	0.10
	4MDBT %	40	–	–	–	–
	2 + 3 MDBT %	34	–	–	–	–
	1MDBT %	26	–	–	–	–

DNR-1 = (2,6 + 2,7)/(1,5)DMN; DNR-2 = (2,6 + 2,7)/(1,4 + 2,3)DMN; TNR1 = (2,3,6)/(1,4,6 + 1,3,5)TMN; MNR = 2-MN/1-MN; MPI-1 = 1.5(2MP + 3MP)/(PHEN + 1MP + 9MP); MPI-2 = 3(2MP)/(PHEN + 1MP + 9MP); MPI3 = (3MP + 2MP)/(9MP + 1MP); MDR = 4MDBT/1MDBT; MPDF = (2MP + 3MP)/(2MP + 3MP + 1MP + 9MP); R_C_(a) = 0.6(MPI-1) + 0.4 (for Ro < 1.3); R_C_(k) = − 0.166 + 2.2424 (MPDF).

## Discussion

### Origin and source of natural gases

The molecular composition (C_1_/C_2_ + C_3_), carbon, and hydrogen isotopes have been applied to investigate the origin of gas following several published works^34–41^**.** In this case, the origin of natural gases in WDDM has been investigated using the most widely used diagrams from the above works.

**Figure 10:**
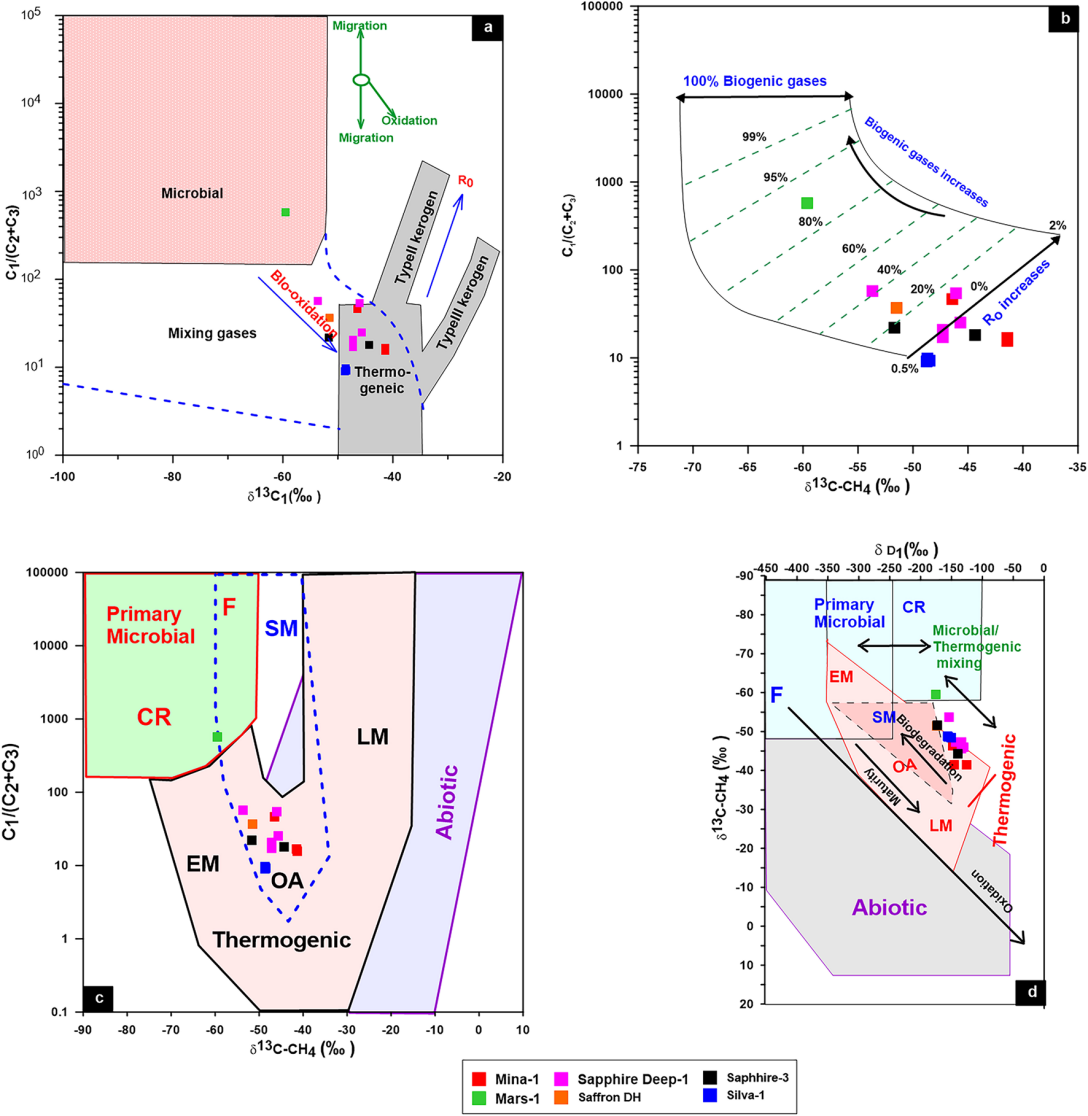
(**a**) The genetic characteristics Bernard diagram of (C_1_/(C_2_ + C_3_) ratio versus δ^13^C_1_; (**b**, **c**) The gas genetic diagrams of^40^; (**d**) the relationship between the ratio of C_1_/(C_2_ + C_3_) and δ^13^C_1_ displays the contribution of biogenic gases as a percentage.

In the current study, most of the studied natural gases in the WDDM have δ^13^C_1_ value lighter than − 30‰ (Table 1), reflecting that these gases are typically biogenic gases rather than abiogenic (hydrothermal origin) following classification of^34^. The biogenic gases can also be classified into bacteriogenic and thermogenic gases. The bacteriogenic gas is characterized by high methane content and high dryness index (> 0.99) with δ^13^C_1_ values lighter than − 55‰ (more negative), while the thermogenic gases have δ^13^C_1_ values heavier than − 50‰ (less negative)^34,39^**.** In this regards, most of the studied natural gases are plotted on the zone of thermogenic gas generation (Fig. 10a-c). However, most of the natural gases in WDDM are unaltered gases, because they mainly belong to the oil associated gas field and away from the field of secondary biodegradation as shown in Fig. 10b,c. There are just two samples from the Sapphire Deep-1 and Mars-1 wells that are microbial to mixing gases (Fig. 10c,d). The high biogenic gases, up to 80%, in the natural gas samples from the Mars-1 well suggest the significant contribution of primary microbial gases (Fig. 10d). These gases result from the initial cracking of organic matter by the action of CO_2_ bacteria (Table 1).

**Figure 11:**
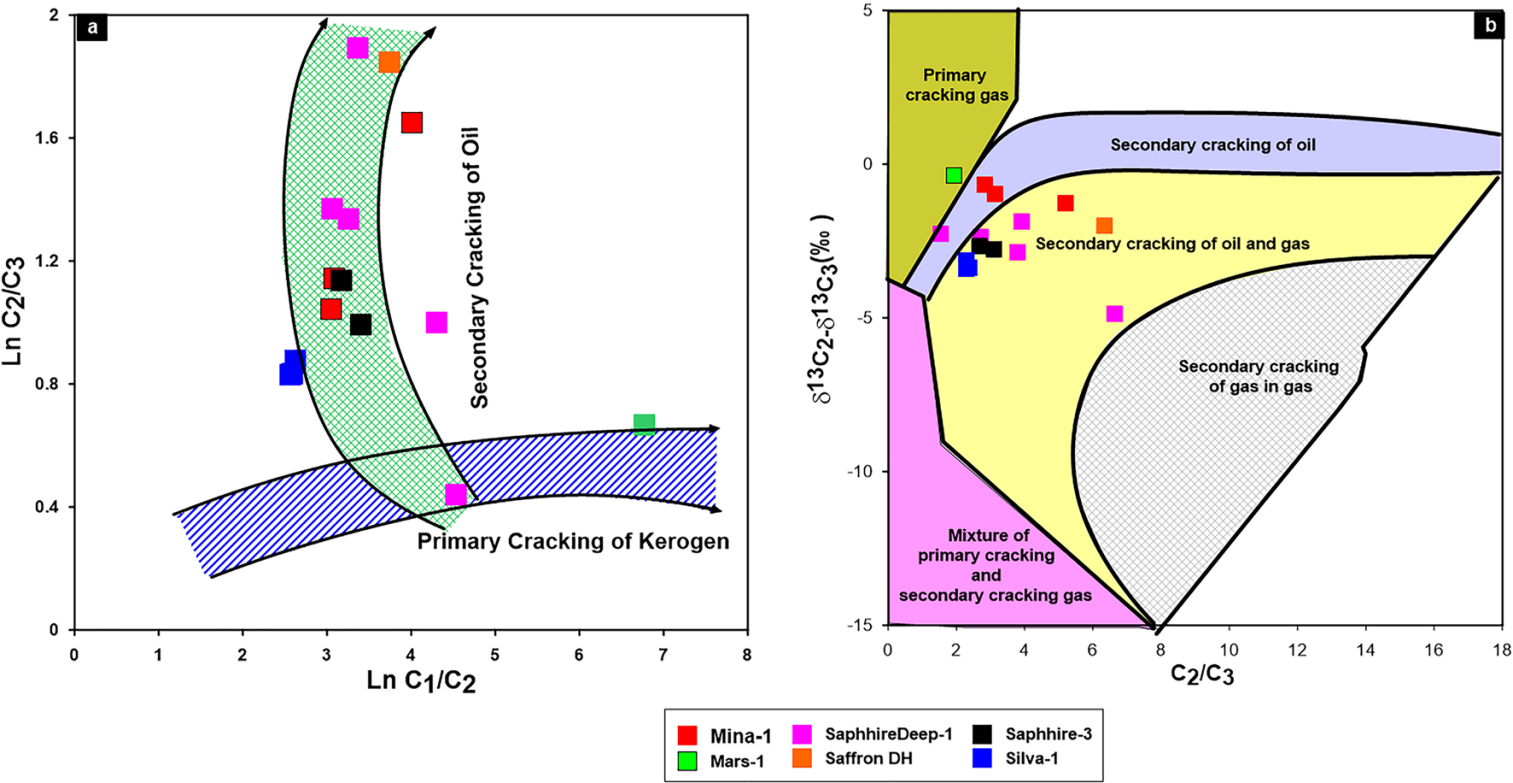
The relations between Ln C_1_/C_2_ and Ln C_2_/C_3;_ and C_2_/C_3_ and δ^13^C_2_–δ^13^C_3_ showing the mechanism of gas generation.

The distinguish between oil cracking gas and kerogen cracking gas for the origin of the natural gases in WDDM has been performed using ln methane (C_1_)/ethane (C_2_) and ln ethane (C_2_)/propane (C_3_) diagram of^42^**.** The ethane/propane ratio (C_2_/C_3_) of oil cracking gas increases rapidly with thermal evolution, whereas that of kerogen-cracking gas remains nearly constant (sometimes declining slightly). Contrarily, the methane(C_1_)/ethane (C_2_) ratio of the oil-cracking gas decreases while that of the kerogen-cracking gas grows and remains essentially constant^36,43^. Accordingly, most of the studied gas samples exhibit a wide range of C_2_/C_3_ values and follow the pattern of cracking oil rather than the primary cracking of kerogen (Fig. 11a), with the exception of one sample from the Sapphire well, which trends to mixing gases formed from both primary and secondary cracking. The biogenic gas sample from the Mars-1 well is mainly formed from primary cracking of kerogen (Fig. 11a). A dominance of the secondary oil and oil/gas cracking was strengthened by the C_2_/C_3_ and δ^13^C_2_–δ^13^C_3_ relationship (Fig. 11b). This relationship also aided in understanding the level of thermal maturity, revealing that most thermogenic gases were formed during the high maturity stages, whereas Mars natural gas is primarily kerogen cracking gas produced by microbial oxidation during the lower maturity stage.

The thermogenic gases can be separated into two groups: those produced from coal-type kerogen (humic), and those that are cracked from oil of types I and II kerogen, and came from marine saprophytic organic matter^44^. Gases with humic source are aromatic in structure and short branched in chain structure, and are relatively rich in ^13^C, while the gases saprophytic source are primarily constituted of long-chain aliphatic structure and are comparatively enriched in ^12^C^34^.

According to empirical observations, the ethane's ^13^C value is frequently employed as an efficient indication to distinguish between gas obtained from coal and gas derived from oil. Coal-type gas’s δ^13^C_2_ and δ^13^C_3_ values were typically greater than − 27.5 and − 25.5, respectively, and oil-derived gas's values were lower than−  29.0 and − 27.0, respectively^45,46^ advocated a δ^13^C_2_ > − 27.5for gases derived from humic kerogen and a ^13^C_2_ > -29 for gases derived from sapropelic kerogen based on a thorough investigation of natural gases in China.

**Figure 12:**
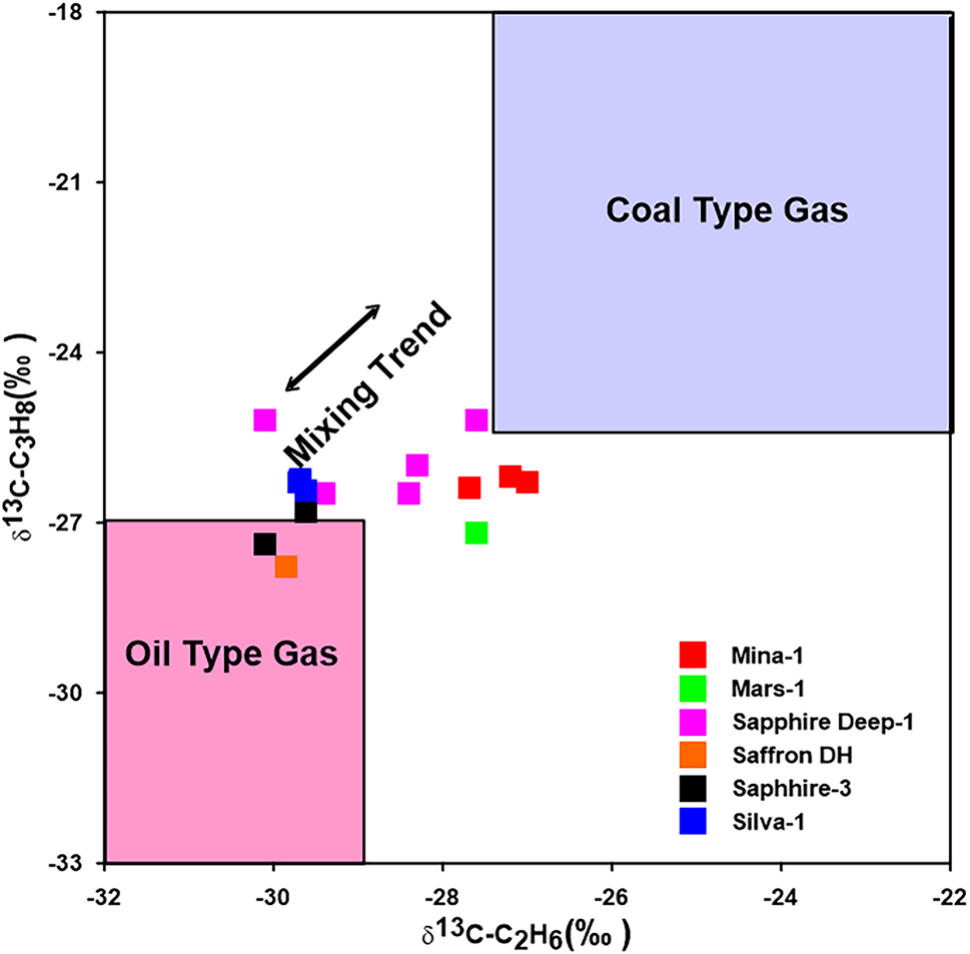
Cross plot of δ^13^C_3_ versus δ^13^C_2_ to distinguish between oil- and coal-type gases.

**Figure 13:**
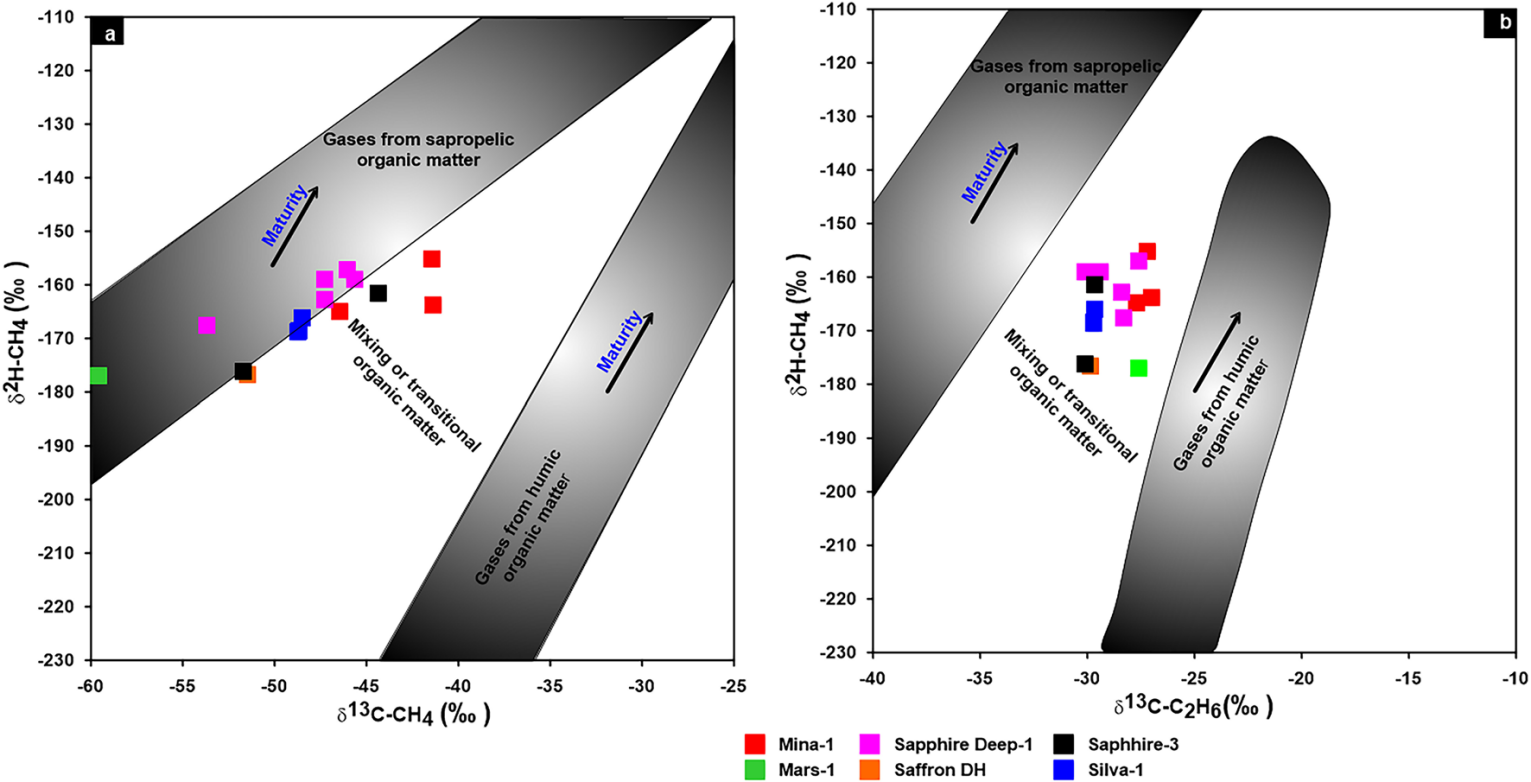
Correlation diagram of δ^13^H-C_1_ versus δ^13^C_1_ and δ^13^C_2_ to distinguish between oil- and coal-type gases.

In the current study, ethane and propane gases show large ranges of ^13^C values in the Miocene gases, with average values of − 28.80‰ and − 26.43‰, exhibiting the characteristics of combining oil/coal type gases (Table 1; Fig. 12). The interpretation of the mixing source of the gas is also demonstrated by the combination between the isotopic composition of natural gas (carbon and hydrogen) i.e., δ^2^HC_1_, δ^13^C_1_ and δ^13^C_2_^34,47–49^. The correlation δ^2^H-C_1_ and δ^13^C_1_ diagram, however, shows that two gases samples from Mars-1 and Sapphire Deep-1 wells are plotted in the zone of sapropelic gases (Fig. 13a), indicating that these gases are formed from low mature sapropelic organic matter that has been blended tiny amount humic organic material. The majority of deeper Miocene gases exhibit the properties of gases produced mostly from the mixing organic matter (Fig. 13b).

**Figure 14:**
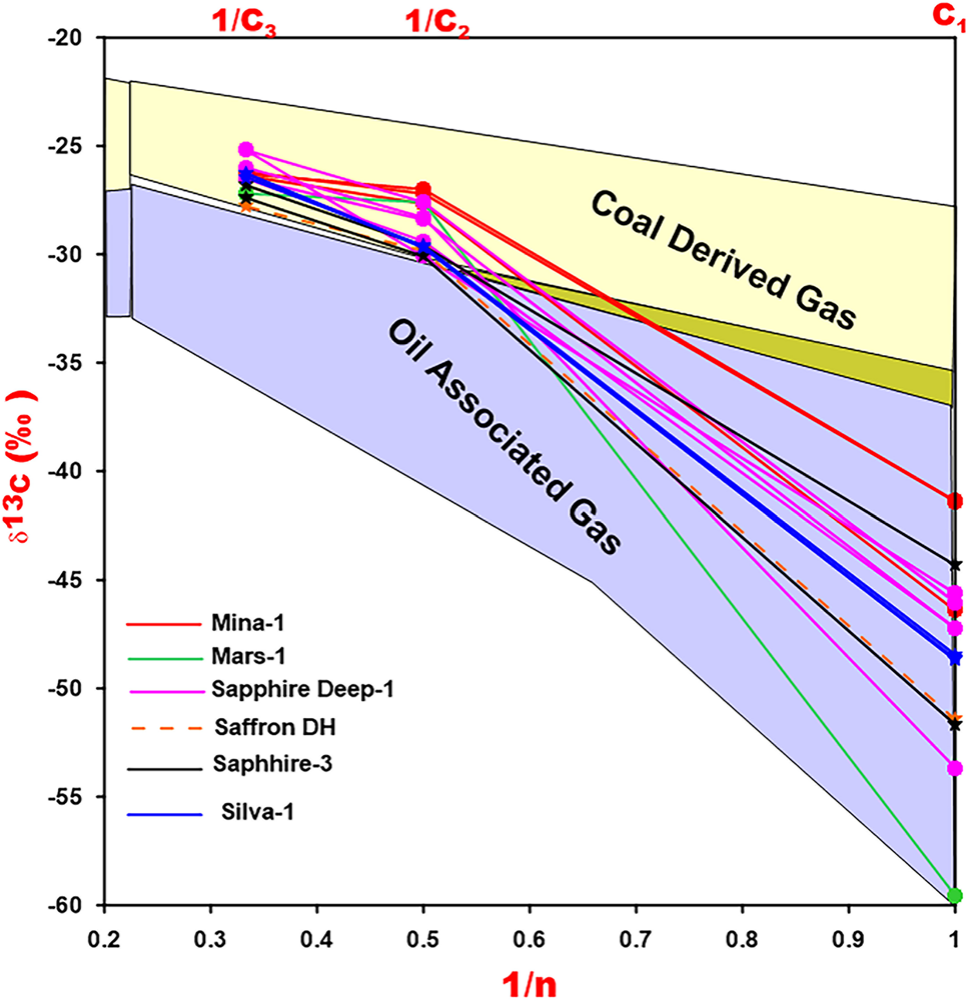
Plot of the WDDM's natural gas data showing the relationship between carbon isotope ratio and the inverse carbon atom number.

The isotopic composition of alkanes (δ^13^C) also supports the interpretation of the gas’s mixing source^50,51^**.** The carbon isotopic values of ethane (C_2_H_4_) and propane (C_3_H_6_) are − 29‰ and − 26.5‰ respectively, are commonly used to differentiate oil type gas from coal derived gas in NGP^51,52^, while^50^ used different boundary values of − 30‰ and − 31‰ respectively. Applying the boundary values of^50^ using natural gas plot (NGP) of δ^13^Cn values and 1/n, most of the studied natural gases show that carbon isotopic pattern of gases [methane (CH_4_), ethane (C_2_H_4_) and propane (C_3_H_6_)] are within the range of mixing source (Fig. 14).

### Origin and source of condensate associated with gases

In this work, the origin and source of condensate was assessed by employing multi geochemical indicators including biomarkers and isotopic composition of saturated and aromatic hydrocarbons (δ^13^C).

The condensate samples also show abundance of low-molecular-weight straight-chain n-alkanes, ranging from about *n*-C_7_ to *n*-C_22_ reflecting marine origin. Only the condensate sample from Silva-1 well displays significant abundance of normal C_20+_ (Fig. 7). With the exception of Silva-1 condensates, which exhibit a high affinity for the non-marine environment, the isotopic compositions of saturated and aromatic fractions (δ^13^C) of condensate samples further point to a heterogeneous depositional environment (transitional environment) (Fig. 6). This explanation is validated by the concentration of the isoprenoids in the gas chromatogram profiles of the studied condensates (Fig. 7).

**Figure 15:**
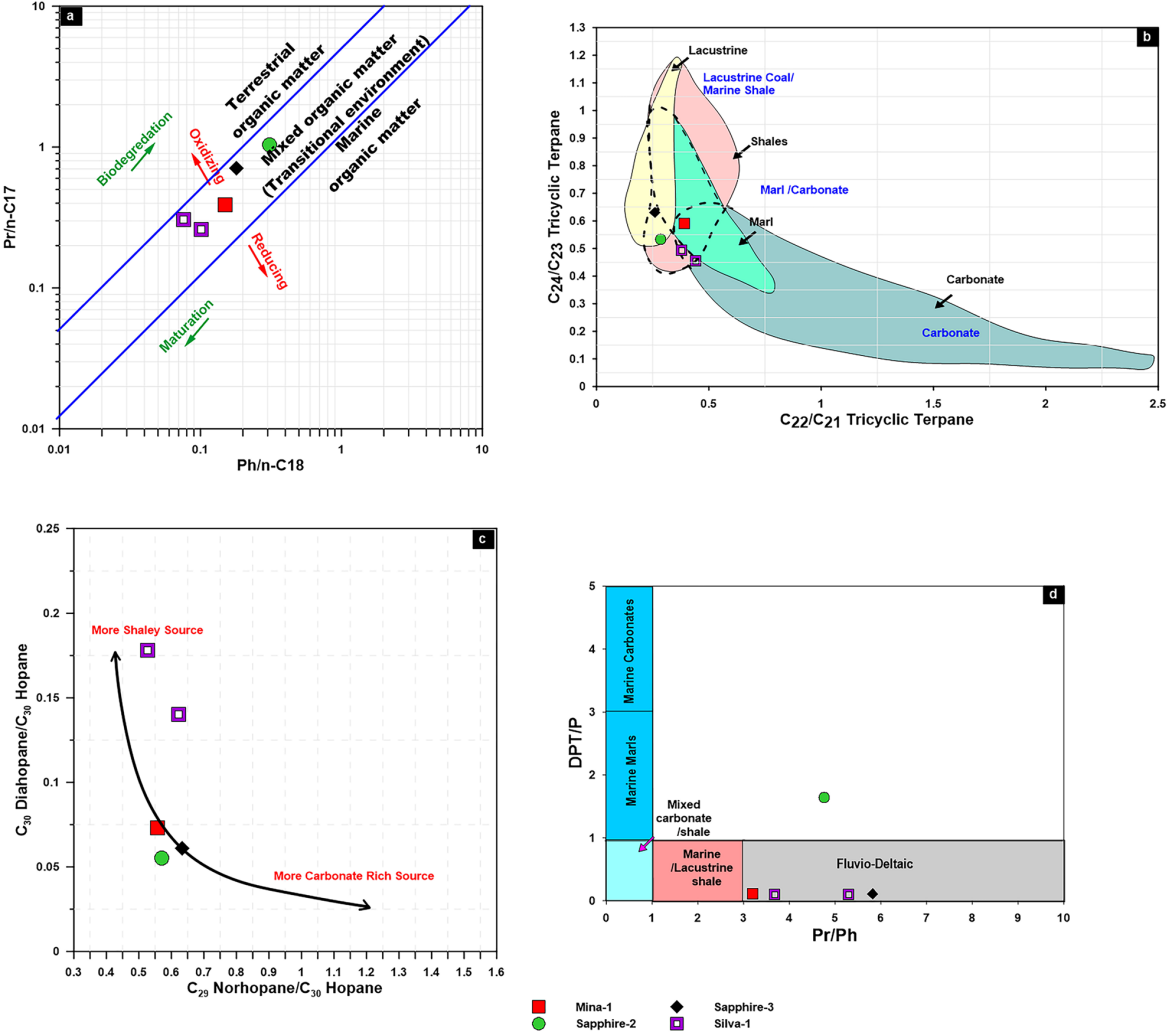
(**a**) Plot of pristane/n-C_17_ versus phytane/n-C_18_ ratios displays the depositional conditions of source rocks of the studied condensate samples; (**b**) Relationships between different tricylic terpanes showing the source facies; (**c**) Cross plot of C_30_ diahopanes/C_30_ hopane showing the source facies; (**d**) Relationship between Pr/Ph and aromatic compound (DPT/P).

The pristane has a greater value than phytane, with pr/ph ratios of more than 3, implying that terrigenous organic matter is the source of the studied condensates. Phytane/*n*-C_18_ and pristane/*n*-C17 ratios, however, suggest that mixed organic materials were deposited under suboxic to somewhat oxic environments under high thermal maturity (Fig. 15a)^2^.

This interpretation of mixed organic matter accumulated is also demonstrated by the environmental setting and source facies. Remarkably, the tricyclic terpane distributions of condensates show abundance of C_19_ and C_20_ tricyclic terpanes with respect to other tricyclic terpanes (Fig. 8a), resulting in relatively low ratios of C_22_/C_21_ tricyclic terpanes (0.26–0.44), and C_24_/C_23_ tricyclic terpanes (0.46–0.63) suggesting clay-rich rock formed in transitional conditions (suboxic) (Fig. 15b). The predominance of C_30_ hopane with respect to C_29_ norhopane, diahopane and other homohopanes (C_31_–C_35_) further indicates clay rich source rock deposited in transitional conditions (suboxic)^53^ (Table 3; Fig. 15c). Similarly, the relative presence of diasteranes in condensate samples suggests a clay-rich source rock^2,8,9^(Fig. 8c).

The presence of the oleanane in the studied condensates is also used as a marker of angiosperm plant input that is associated with deltaic environment (Fig. 8a). However, the higher plant material found in deltaic systems typically contains no or little tricyclic terpanes, with the C_19_ and C_20_ homologs being the most abundant^53^**.** By contrast, marine and lacustrine organic matters show abundance of the C_20_ homologs.

The presence of gammacerane in low concentration together with low C_35_/C_34_ homohopanes ratios(0.38–0.84) also suggests organic matter formed in a freshwater environment under suboxic conditions (Fig. 8a; Table 3)^54^. However, the low salinity stratification in the source rock of the studied condensates may be attributed to the dilution of marine water caused by the entry of freshwater from the proto-nile river^4^.

In addition, the aromatic sulfur compounds identified in the studied condensate have also been used to infer depositional conditions and lithology of the rock generated the studied condensate. Dibenzothiophene (DBT) and their methyl homologs are commonly employed to assess the lithology and environmental setting of the source rock^55^. The relation between Dibenzothiophene (DBT)/Phenanthrene and pr/ph ratios of^55^ was used to conclude the depositional environment of studied condensates and suggests that relatively all studied condensates sourced from organic matter formed in fuvio-deltaic environment (Fig. 15d).

**Figure 16:**
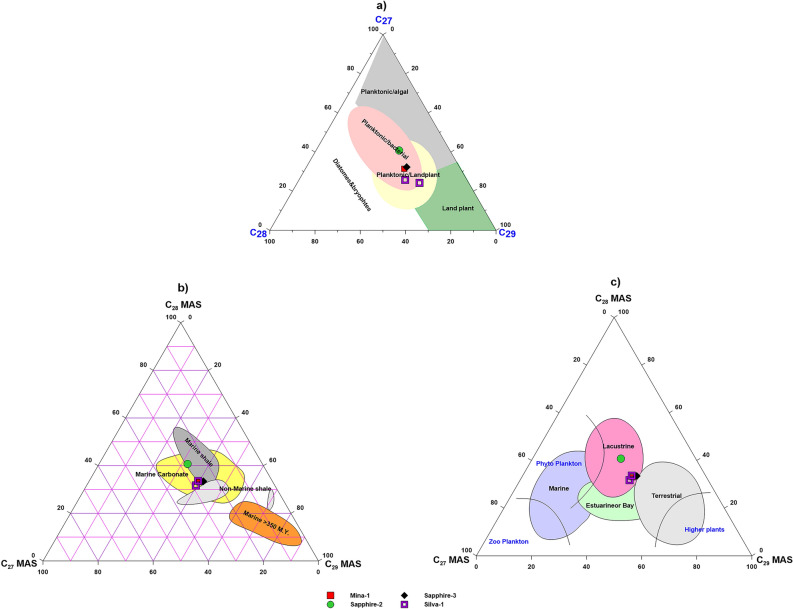
(**a**) Ternary diagram of C_27_, C_28_ and C_29_ regular steranes and (**b**, **c**) Ternary diagrams of monoaromatic steroids (MAS) showing source of the organic matter.

Using sterane biomarkers, the precursors of organic matter for the analyzed condensates were investigated further (Whether its origin is algae, land plant or bacteria). The higher abundance of the C_29_ regular sterane than C_27_ and C_28_ regular steranes of the examined condensate samples (Table 3 and Fig. 8b,c), supporting the explanation of the contribution of mixture marine/non marine organic matter formed in transitional environment based on the adapted Huang and Meinschein ternary diagram (1979) as shown in Fig. 16a^56^. Low steranes/hopanes ratios (0.32–0.44) also contribute to this mixing organic matter (Table 3), since low sterane/hopane ratio is typical indicative for contributing mixed organic matter and a higher microbial activity during organic matter deposition^53,57^. The existence of C_30_ steranes in some condensates indicates the presence of chrysophyte marine algae (1.99–3.11) (Table 3; Fig. 8c).

Numerous of heterocyclic and polycyclic aromatic HCs are more abundant in all oil and mature source rocks^58^ and are useful indicators of organic facies type, depositional environment and lithology^59^.

The distribution of dimethyl- and trimethyl-naphthalenes in the aromatic HCs have been widely employed as indicators of higher plant input^60^. The input of angiosperm and gymnosperm to the organic matter is evidenced by the occurrence of 1, 2, 5- and 1, 2, 7-trimethylnaphthalenes^61,62^. The presence of 1,2,7-TMN has been found in high concentrations in crude oils and source rocks of terrestrial organic origin., while the oils generated from marine source rock have 1, 2, 7-/1, 3, 7- and 1, 2, 7-/1,2,6-trimethylnaphthalenes (TDE) ratios less than 1^63^. However, the organic matter with 1, 2, 5-/1, 3, 6 trimethylnaphthalene greater than 0.30 originated mainly from non-marine materials. In this case, although the studied condensate sample has high 1, 2, 5-/1, 3, 6 trimethylnaphthalene (> 0.30,  = 0.66), it also has low 1, 2, 7-/1, 3, 7- and 1, 2, 7-/1,2,6-trimethylnaphthalenes ratios of less than 1 (Table 4), indicating mixed source of organic matter.

Moreover, the distributions of alkylated phenanthrenes and naphthalenes in the aromatic HCs, with the ratio of 1-methylphenanthrene (1 MP) and 9-methyl Phenanthrene (9 MP) are frequently used as organic matter type indicators. Elevated abundance of 9-MP has been observed in sediments of marine origin while high amounts of 1-MP is found in sediments with higher plant origin^64^. The 9-MP is generally abundant, with significant amounts of 1-MP in the Mina condensate (Fig. 9e), with 1-MP/9-MP ratio close to 1 (0.83 as see in Table 4), indicating a marine organic matter with notable amounts of land plant inputs^65^**.**

This interpretation is further supported using the distribution of monoaromatic steroids (C_27_–C_29)_ and their percentages (Table 4). Compared to steranes, the higher abundance of the C_28_ and C_29_ monoaromatic steroids than the C_27_ of the examined condensate sample (Table 4 and Fig. 9h), suggests that this condensate was generated from non-marine shale source rock that was deposited in transitional environments based on the adapted ternary diagrams (Figs. 16 b-c)^65^.

### Estimation maturity levels of natural gases and associated condensate

Natural gas maturity was estimated using the δ^13^C of CH_4_, C_2_H_4_ and C_3_H_6_ (methane, ethane and propane) gases. The kinetic isotope effect refers to the different behaviour of ^12^C and ^13^C atoms during breakages of the chemical bond between two carbon atoms(C–C)^66^.

**Figure 17:**
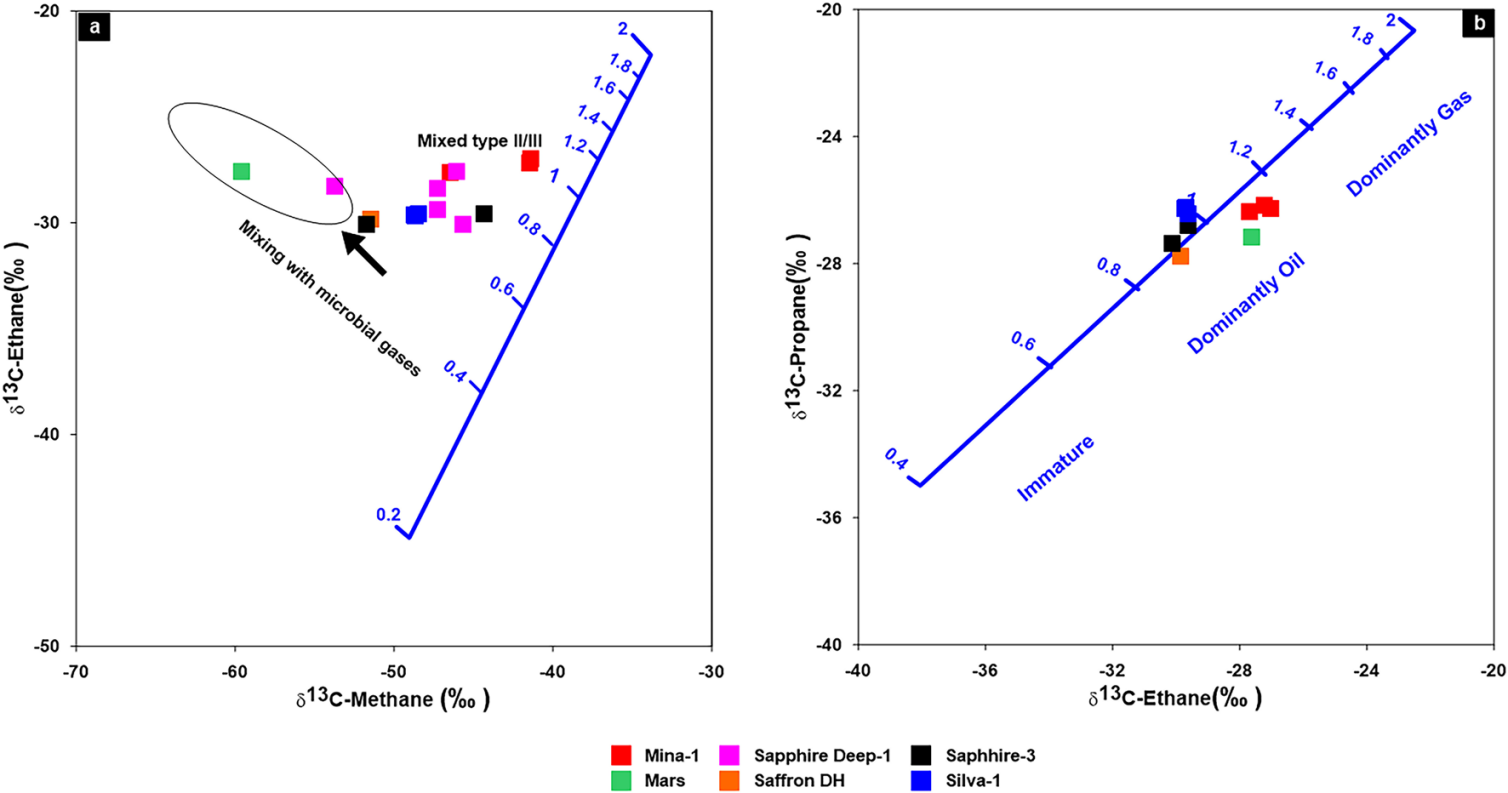
Isotopic maturity models of the studied gases using^66^based on δ^13^C_kerogen_.

In this study, most of the natural gases from the studied wells in WDDM are mainly formed from mixed sources as highlighted in the previous subsections (see Figs. 12, 13, 14). Therefore, the diagnostic cross plots of carbon isotope data of the mentioned gases were used and provided valuable information about the maturity of mixed gases and the related source rock maturities based on the adapted diagram of^66^. These plots reveal that all samples plot away from the maturity line of type II, reflecting the mixing source of these gases, with maturity values (R_O_) ranging from 0.5 to 1% (Fig. 17a). Most of the natural gases were mainly sourced from mixed organic matter and generated at a more mature stage than the other two gas samples from Mars and Sapphire Deep-1 wells (Fig. 17a). On the other hand, the carbon isotopic composition of heavy hydrocarbon gases (C_2_H_4_ and C_3_H_6_) are inextricably linked to the source rocks and better allows predicting gas maturity and their sources^66^. The isotopic composition of ethane and propane in most of the natural gases already clarify their derivation from mixed kerogen type II/III at maturity degrees ranging between 1% and 1.2% (Fig. 17b). This result evidently suggests that most of the thermogenic gases in the WDDM represent a migration of wet and dry gases upward from pre-Miocene source rocks with high maturity levels in the range of 0.8–1.2%. These values are generally consistent with a wide range of maturities ranging from 1.0% to 1.5% for natural gases derived from Type-II or Type-II/III kerogens, as reported by previous work^8^.

In addition to natural gases, saturated and aromatic HC fractions can also be utilized as maturity indicators to measure the maturation levels of the studied condensates^54^.

**Figure 18:**
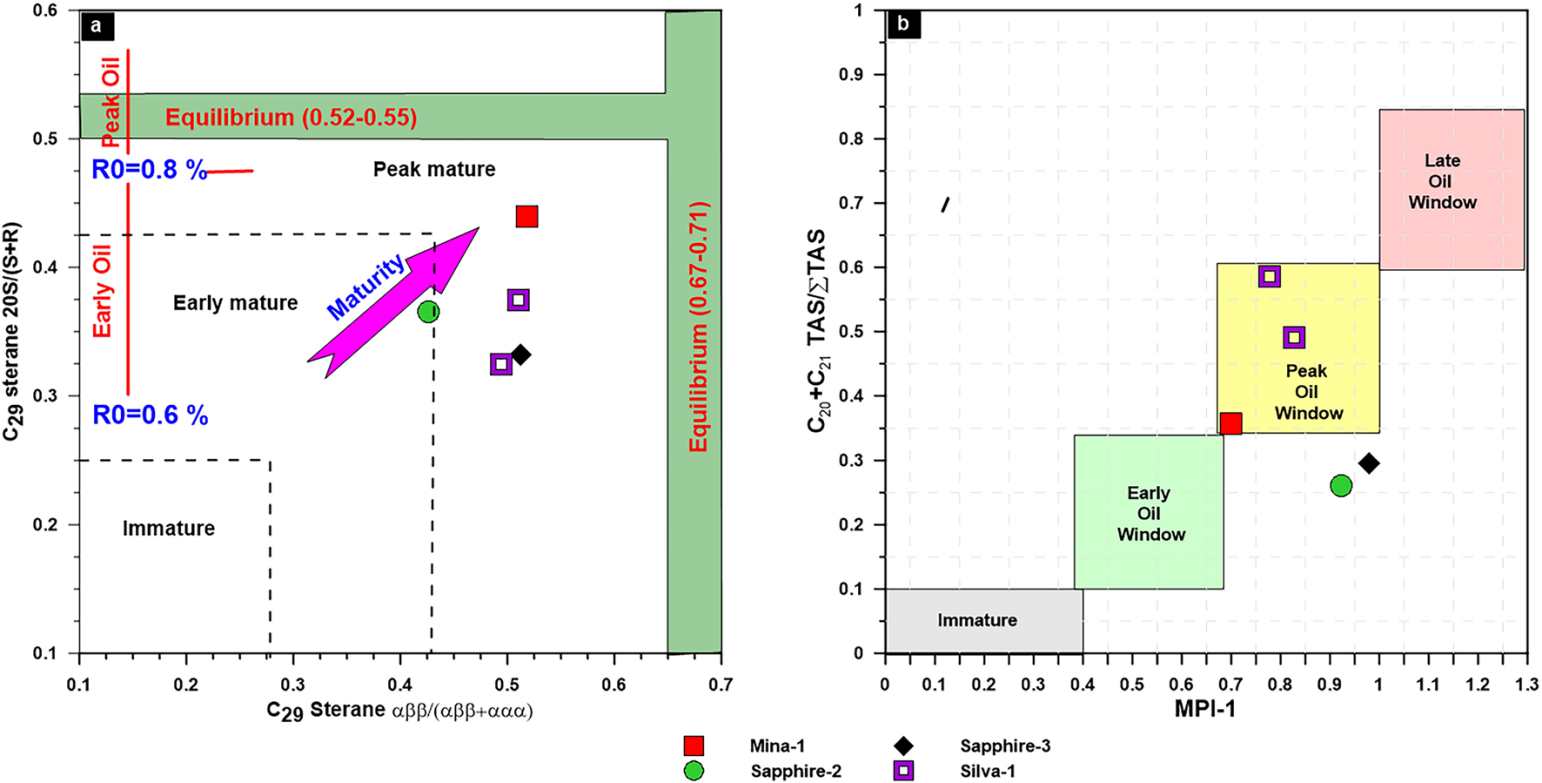
Plots of maturity-related biomarker ratios demonstrate qualitative hydrocarbon yield showing the relation between two main sterane isomerization C_29_ 20S/(20S + 20R) and C_29_ (ββ 20S/(ββ 20S + αα20R) and the relation between MPI-1 and C_20_ + C_21_/∑TAS (Triaromatic steroids).

The most accurate indications of biomarker maturity were terpanes and steranes, specifically 22S/(22S + 22R) in C_32_ homohopanes, and 20S/(20S + 20R) and ββ/(ββ + αα) in C_29_ steranes (Fig. 8) (For more information, see Table. 3). The thermal maturation of the crude oil and source rock samples can be assessed using the C_32_ homohopane ratio. This ratio rises to a maximum of 0.70 as thermal maturity increases^53^. The organic matter of the source rock is considered immature if the C_32_ ratio falls below 0.50. C_32_ ratios of 0.50 to 0.58 are found in early to moderately mature source rocks, whereas an equilibrium point greater than 0.58 indicates the peak oil window and subsequent maturation phases^67^. Following this scale, the examined condensate reached equilibrium with a C_32_ hopane ratio value 0.59 (Table 3), reflecting that the condensate produced from source rocks has progressed to the major oil generation phase. It is also important to note that the ratios of the C_29_ sterane 20 S/(20S + 20R) and ββ/(ββ + αα) ratios show the thermal maturity of the crude oil samples^67^. These ratios reached to the equilibrium in the studied condensates, and suggesting that the studied condensate was formed at high maturity stage at the peak of oil generation as shown by combining the C_29_ sterane 20 S/(20S + 20R) and ββ/(ββ + αα) ratios (Table 3; Fig. 18a). Furthermore, to assess the organic matter maturity, multiple biomarker maturity ratios and parameters of the methylated phenanthrene and naphthalene distributions in the aromatic HC fraction of the analysed condensate sample can be used^68–72^ These aromatic maturity parameters are presented in Table 4 and include methylphenanthrene ratio (MPR) methylphenanthrene index (MPI), alkyldibenzothiphene parameter (MDR), methylnaphthalene ratio (MNR), dimethylnaphthalene ratio (DNR) , trimethylnaphthalene ratio (TNR) and triaromatic steroids (TAS). The methylphenanthrenes and alkyldibenzothiphene show the dominant abundance of 3-MP and 2-MP in m/z 192 mass fragmentograms, while 4-MDPT is a more abundance in the m/z 198 mass fragmentograms (Fig. 9).

The abundance of phenanthrene in hydrocarbons or source rocks is generally affected by increasing the thermal maturity. The concentrations of 2- and 3- methylphenanthrene increase more than those of 1-and 9-MP with increasing maturity^69^. The methylphenanthrene index (MPI) is one of the most widely used parameters used to evaluate thermal maturity based on the relative abundance of phenanthrenes and their homologs^73^. Calculated the vitrinite reflectance (Rc_(rw)_) from MPI-1 based on the best correlation between MPI-1 values and measured vitrinite reflectance in coal samples. However^74^, estimated the coal maturity(Rc(k) based only on the distribution of the mono-methylphenanthrenes, not on the relative abundance of the phenanthrenes. He developed the Methylphenanthrene distribution factor (MPDF) as a maturation indicator.

The MPI-1, MPI-2, and MPI-3, values of the studied samples were estimated and found to be in the range of 0.70–1.07 (Table 4). These high values of methylphenanthrene index (MPI-1) and alkyldibenzothiphene (MDR) of the analyzed oil samples show equivalent vitrinite reflectance (Rc_(rw)_) and (Rc_(k)_) in the range of 0.80–1.0%, respectively (Table 4), implying mature source rocks (peak to late mature of the oil generation window)^73,74^. This finding is demonstrated by the predominance of 1, 2, 5-trimethylnaphthalene (Table 4; Fig. 9), with respect to 1, 2, 7-trimethylnaphthalene is typical of mature oil characters^60,75^.

Monoaromatic and triaromatic steroids were also utilised to determine thermal maturity since they were more resistant to the effects of biodegradation than alkane-type biological indicators^76^. The high values of C_20_ + C_21_)/Σ TAS of the analyzed condensates, signifying the high maturity stage. In combining the ratios of MPI-1 and triaromatic steroids into one relationship, as illustrated in Fig. 18b, this result is seen.

Additionally, the Table 4 ratios of dimethylnaphthalenes (DNR), methylnaphthalene (MNR), and trimethylnaphthalene (TNR-2) support the high thermal maturity of the analysed oil samples. The studied condensate shows a high MNR (2-methyl naphthalene/1-methylnaphthalene) more than 1 (= 1.6; Table 4) which implies that the high-mature condensate originated from high mature organic matter^76^. Further, the ratio of dimethylnaphthalenes (DNR) increases as thermal maturity increases. depending on the relative abundance of β isomers (2,6- and 2,7) dimethyl naphthalenes with respect to α 1,5-dimethyl naphthalenes. The source rocks are classified into immature source rocks with DNR values less than one, moderately mature source rocks with DNR values between one and three, and highly mature source rocks with values greater than three. The DNR value for the studied condensate is > 3 (Table 4), suggesting a high mature sample reached the peak of oil generation^76^.

The trimethyl naphthalene ratio (TNR) which is represented by the ratio of (2, 3, 6)/(1, 4, 6 + 1, 3, 5) TMN, is considered one of the most accurate indicators of thermal maturity^77^. ^77^ consider that the TNR value of less than 0.5 is related to immature source rocks, while, the TNR value of more than 0.5 is related to mature source rocks. As the studied condensate has a TNR value garter than 0.5, mature is a phrase that refers to the studied condensate.

The abundance of methylbiphenyls has also been developed to evaluate the maturity levels of hydrocarbons and organic matter^78^. The ratio of 3-methylbiphenyl (MBP)/(3- + 4-) methylbiphenyl (MBP) can be used as a maturity indicator because 3- and 4- methylbiphenyls are more abundant than 2-methylbiphenyl (MBP) as thermal maturity increases^78^. The studied condensate has 3- methylbiphenyl (MBP)/(3- + 4-) methylbiphenyl (MBP) ratio equal to 0.7 indicating high maturity.

### Geochemical correlations (gas–gas, gas-condensate and gas/condensate-source rock correlation)

In order to investigate gas–gas correlation variables, this study used molecular and isotope compositions of natural gases. The evaluation of these natural gases as a result of gas–gas correlation shows that the fifteen gas samples studied in the present paper could be grouped into two families. The source organic matter, maturity-related metrics described in the preceding subsection, and cross-plots between these natural gases support these two conceptually distinct categories (Figs. 3, 10–13). Indeed, thirteen gas samples can be incorporated in a single family, while other two gases from the Mars-1 and Sapphire Deep-1 wells can be recognized as a different family. This finding is claimed from the distribution of molecular and isotope features (Figs. 3, 10–13). In this case, most of the natural gases are thermogenic methane gases (Figs. 3, 10a–c) formed from secondary cracking of oil and oil/gas (Fig. 10). The probable source rock for these thermogenic methane gases contains mixed organic matter (Figs. 12, 13, 14). In addition, these thermogenic methane gases were formed at higher thermal maturity stages than the other gases from the Mars-1 and Sapphire Deep-1 wells, equivalent to a maturity range between 0.80 and 1.2% (Fig. 17).

The group II family, which consists of two gas samples from the Mars-1 and Sapphire Deep-1wells, has relatively low molecular compositions and hydrogen and carbon isotopes values (δ^13^C_1_-C_3_) values compared to other natural gas samples from group I (Table 1). This suggests that most of the gases in the group II family are bacteriogenic gases that formed from primary cracking of kerogen (Fig. 10) at relatively low thermal maturity (Fig. 17a). This finding of the high contribution of primary bacteriogenic gases of the natural gas samples from Sapphire Deep-1 and Mars wells is supported by the distribution of biogenic gases, reaching up to 40 and 80%, respectively (Fig. 10d) and high amounts of CO_2_ gases in these two natural gases (Table 1). These biogenic gases were formed from a mixed organic matter, with a high input of sapropelic materials (Fig. 13a).

The different genetic gas families indicate their derivations from different sources with varying degrees of thermal maturity. This finding is consistent with previous study which suggested that the Miocene biogenic source is charging Serravallian reservoirs, while the Oligocene biogenic to thermogenic source is charging Aquitanian-Chattian reservoirs in the Eastern Nile delta^79^. Likely, research studies on the same study area documented that none of the Miocene and Oligocene formations reached the stage of peak oil or gas generation, indicating immature to maturity near the beginning of the oil window^2,4^. Conclusively, we assume that the low mature Miocene and Oligocene formations are the source of biogenic gases in Western Nile delta.

On the same hand, the source of the thermogenic gas is still unidentified. To address this point in this study, we discussed the source organic matter and maturity-related biomarker parameters of the saturated and aromatic HCs for the analysed condensates and the isotopic signatures of natural gases, and investigated the correlations between these condensates and associated thermogenic gases in the studied wells. Interestingly, both condensate and associated thermogenic gases are generated from clay-rich units containing organic matters. These organic matters are rich in kerogen type II/III and are generated at high thermal maturity levels. These features are closely related to the Pre-Miocene source rocks (Jurassic–Cretaceous) as evidenced by age dating biomarker results of condensate samples.

Age-specific biomarkers are powerful tools to date the age of hydrocarbons, which is applying individual age-specific biomarkers that give an indication of the age of the possible source rock^4,80,81^.

In this study, the oleanane index and C_28_/C_29_ββS sterane ratio were used to estimate the age of the condensates and associated natural gases' possible source rock^81^, however, reported that oleanane is an indicator of the presence of angiosperm plants and suggested that the low oleanane index is reported in the source rock deposited during the Early Cretaceous and giving rise to high concentration during the Tertiary time with oleanane index value of more than 0.3.^80^ also stated that the C_28_/C_29_ββS sterane ratio increases with decreasing source age of oils. Accordingly, the low C_28_/C_29_ββS sterane (< 0.5) is recorded for Early Palaeozoic or older, while values between 0.4 and 0.7 are recorded for the Late Palaeozoic to Early Jurassic oils, and high ratio of more than 0.7 is recorded for Late Jurassic–Miocene oils.

**Figure 19:**
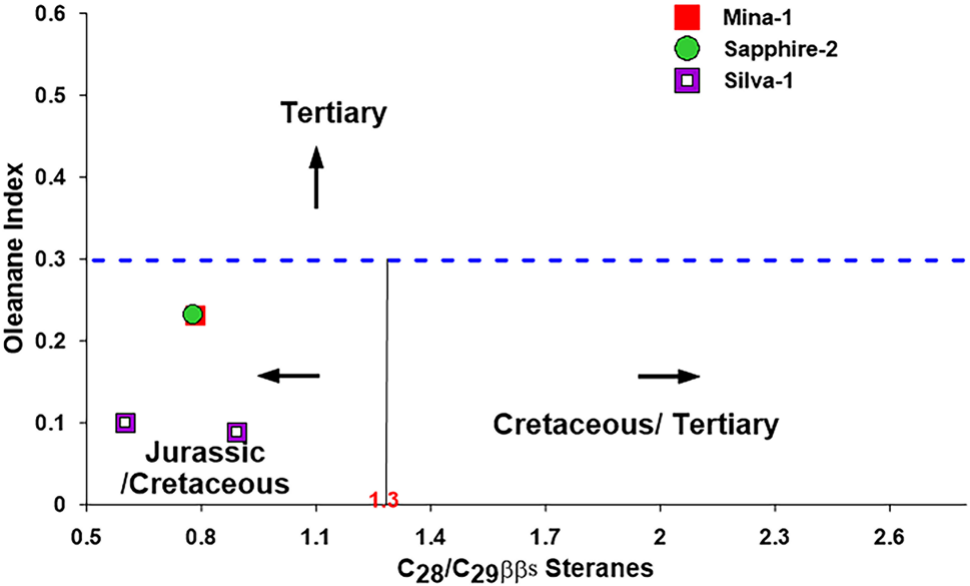
Cross plot of the oleanane index vs. the ratio of C_28_ββS/C_29_ββS steranes^80^.

In this study, the age of the analyzed condensate sample was evaluated based on combining these two indicators and suggested that the condensate samples was generated from source rocks, ranging in age from Jurassic–Cretaceous as demonstrated by the cross-plot of the oleanane index and C_28_/C_29_ββS sterane ratio (Fig. 19).

This age dating biomarker results of condensate in the WDDM correspond to the source rock characteristics of the potential source rock in the Nile Delta and in the nearby Northern Sinai hydrocarbon provinces reported by previous works^4,82^. The late Mesozoic (Jurassic–Cretaceous) and Cenozoic (Oligocene–Pliocene) sedimentary successions in Nile Delta and the nearby Northern Sinai hydrocarbon provinces have a variety of oil/gas-prone source rocks^3,4,82,83^. These source rocks are mainly clay-rich and have kerogen types -III and II/III^4,82^.

The main source rocks of gas prone, with primarily type III kerogen, are thought to be the Oligocene to Early Miocene units in the Nile delta^2–4,9^. In contrast, the Jurassic to Cretaceous sedimentary succession has a variety of oil/gas-prone source rocks, with mainly mixed kerogen types (II/III) in the nearby Northern Sinai province^82^. However, the Jurassic to Cretaceous units are anticipated to exist offshore of northern Egypt and potentially buried deeply beneath the Nile Delta (Fig. 2).

Furthermore, using 1-D basin models, researchers are investigating the maturity and history of hydrocarbon generation in potential late Mesozoic and Cenozoic source rocks in the Nile Delta and nearby Northern Sinai provinces^82,84^. By Calculating the total petroleum expulsion in relation to the total hydrocarbon in-place (discovered) shows the presence of additional source rock (Jurassic) is required in addition to the Oligocene Miocene source rock,whereas the total amount of hydrocarbon discovered until now exceeds the Oligo-Miocene source rock capabilities^84^.

**Figure 20:**
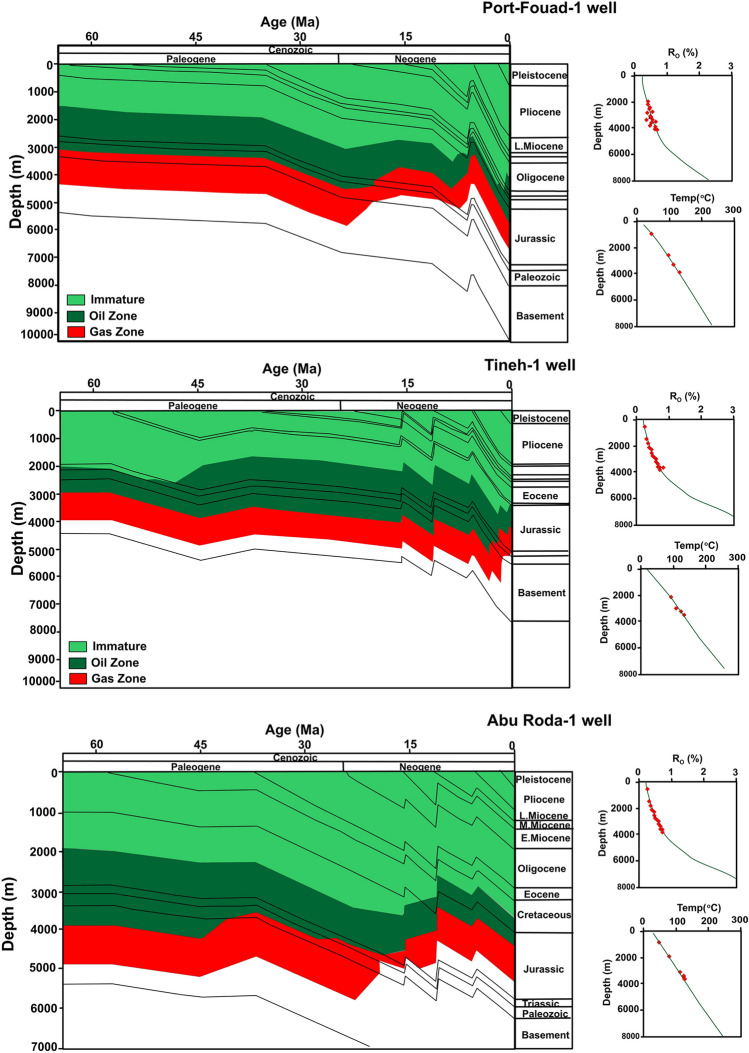
Burial overlap with thermal maturity history (colored areas) cross all rock units (left) and optimized fit of calibrated data i.e. bottom-hole temperatures (BHT) and measured vitrinite reflectance (%VR)] and models of EASY %Ro maturity and geothermal (right).

According to basin models^82^, the Jurassic and Cretaceous units are buried approximately 2800–65,000 m deeper than the Cenozoic formations, and reached a main oil- and gas generation window (Fig. 20), indicating that the Jurassic and Cretaceous units are mature and genetically linked enough to be effective source rocks in the nearby Northern Sinai province. The basin models also show that the Jurassic source rocks are thermally more mature than the Cretaceous source rocks and have reached a gas window and a relatively high thermal maturity level (Fig. 20), causing most of the oil from the Jurassic source rocks to be cracked into condensate (wet gas) and then natural gases. Therefore, the organic-rich intervals of Jurassic units in the nearby Northern Sinai are provinces considered the main source rock of the Miocene condensate thermogenic methane gases in the Nile Delta, and these natural gases are non-indigenous and migrated from higher mature source rock from the nearby Northern Sinai provinces or from the Jurassic formations that were anticipated from the offshore Nile Delta basin (Fig. 2).

## Conclusion

Fifteen natural gases and five condensate samples taken from different well locations in the WDDM were geochemically investigated and utilized to define the origin of the natural gases and condensate accumulations and the characteristics of their probable source rocks, including organic matter (OM) origin, depositional conditions, lithology, and thermal maturity.

The study of the molecular compositions and carbon/hydrogen isotopes reveals that there are various types of gases in the WDDM, mainly thermogenic methane gases, with small contributions of biogenic methane gases. Both thermogenic and biogenic methane gases originated from mixed organic matters, showing high continuations of the sapropelic organic matter for biogenic gases origin. Most of the natural gases in WDDM were created by the secondary cracking of oil and oil/gas and are considered to be thermogenic gases. In contrast, the biogenic gases are formed from the primary cracking of kerogen at the low maturity stage by the action of bacterial CO_2_ reduction, as demonstrated by the high amounts of CO_2_ gases.

The biomarker signatures of the Miocene condensates display their origin as mixed organic matter deposited in a fluvial-deltaic environmental setting under relatively less reducing-more oxidizing conditions (suboxic). The source organic matter and maturity-related biomarkers, together with the age dating markers, suggest that this condensate and associated natural thermogenic methane gases originated from the same source rock, ranging from Jurassic to Cretaceous in age (Khatatba-Abu Roash fms) and generated from mixed organic matter types at different maturity stages.

Most of the natural thermogenic methane gases together with the associated condensate primarily sourced from mature Jurassic intervals and formed by secondary oil and oil/gas cracking at the gas generation window as demonstrated by the basin models of the hydrocarbon generation history. Therefore, most of the natural gases in WDDM are non-indigenous and migrated from more mature Jurassic source rocks (Khatatba Fm) in the nearby Northern Sinai provinces or the deeper sequences in the offshore Nile Delta provinces.

## Data Availability

The datasets used and/or analysed during the current study available from the corresponding author on reasonable request.

## Abbreviations

N: Naphthalene
MN: Methylnaphthalene
EN: Ethylnaphthalene
DMN: Dimethylnaphthalene
EMN: Ethylmethylnaphthalene
TMN: Trimethylnaphthalene
TeMN: Tetramethylnaphthalene
P: Phenanthrene
MP: Methylphenenthrene
DMP: Dimethylphenenthrene
MBP: Methylbiphenyl phenanthrene
MDBT: Methyldibenzothiophene
TAS: Triaromatic steroids
MAS: Monoaromatic steroids
